# KDM1A promotes tumor cell invasion by silencing TIMP3 in non-small cell lung cancer cells

**DOI:** 10.18632/oncotarget.8563

**Published:** 2016-04-02

**Authors:** Lingzhi Kong, Peng Zhang, Wang Li, Yan Yang, Ye Tian, Xujun Wang, Sujun Chen, Yuxin Yang, Tianhao Huang, Tian Zhao, Liang Tang, Bo Su, Fei Li, X. Shirley Liu, Fan Zhang

**Affiliations:** ^1^ Clinical Translational Research Center, Shanghai Pulmonary Hospital, Tongji University School of Medicine, Shanghai 200433, China; ^2^ Department of Thoracic Surgery, Shanghai Pulmonary Hospital, Tongji University School of Medicine, Shanghai 200433, China; ^3^ The Central Laboratory, Shanghai Pulmonary Hospital, Tongji University School of Medicine, Shanghai 200433, China; ^4^ School of Life Science and Technology, Tongji University, Shanghai 200092, China; ^5^ Department of Biology, New York University, New York, NY 10003, USA; ^6^ Department of Biostatistics and Computational Biology, Dana-Farber Cancer Institute and Harvard School of Public Health, Boston, MA 02215, USA

**Keywords:** KDM1A, H3K4me2, TIMP3, JNK, non-small cell lung cancer

## Abstract

Epigenetic regulation plays an important role in tumor metastasis. KDM1A is a histone demethylase specific for H3K4me2/me1 demethylation, and has been found to be overexpressed in many cancers, including non-small cell lung cancer (NSCLC). However, the role of KDM1A in lung cancer remains unclear. Here, we show that KDM1A promotes cancer metastasis in NSCLC cells by repressing TIMP3 (tissue inhibitor of metalloproteinase 3) expression. Consistently with this, overexpression of TIMP3 inhibited MMP2 expression and JNK phosphorylation, both of which are known to be important for cell invasion and migration. Importantly, knockdown of TIMP3 in KDM1A-deficient cells rescued the metastatic capability of NSCLC cells. These findings were also confirmed by pharmacological inhibition assays. We further demonstrate that KDM1A removes H3K4me2 at the promoter of TIMP3, thus repressing the transcription of TIMP3. Finally, high expression of KDM1A and low expression of TIMP3 significantly correlate with a poor prognosis in NSCLC patients. This study establishes a mechanism by which KDM1A promotes cancer metastasis in NSCLC cells, and we suggest that KDM1A may be a potential therapeutic target for NSCLC treatment.

## INTRODUCTION

Non-small cell lung cancer (NSCLC) is one of the most common cancers and is the leading cause of death from cancer in men worldwide [1]. Mechanisms of epigenetic regulation, such as histone methylation, play an important role in tumorigenesis and metastasis [2–4]. How epigenetic dysregulation promotes the gain of tumorigenicity, for example, in NSCLC cells, remains unknown.

KDM1A, also known as LSD1 or AOF2, specifically demethylates mono- and dimethylated H3K4 (H3K4me2 and H3K4me1) [5, 6]. It functions as a transcriptional co-repressor within the repressive chromatin complex, which includes Co-REST as well as HDAC1/2 [7–9]. Overexpression of KDM1A contributes to tumorigenesis in many types of cancer [10]. In particular, it has recently been shown that overexpression of KDM1A promotes proliferation, migration and invasion in NSCLC cells [11]. However, key target genes and signaling pathways that are regulated by KDM1A in NSCLC remain poorly understood.

Extracellular matrix (ECM) remodeling plays an active role during cancer metastasis [12, 13]. Matrix metalloproteinases (MMPs) and tissue inhibitors of metalloproteinases (TIMPs), which are localized in the ECM, regulate ECM homeostasis and remodeling. MMPs can degrade most ECM proteins and regulate the activities of cytokines, cell receptors, and growth factors within the ECM [14]. There is a positive correlation between elevated MMP levels within the tumor stroma and tumor cell invasion or metastasis [15]. However, TIMPs can bind to enzymatically active MMPs and inhibit MMP activity. The TIMP family has four members, TIMP1, TIMP2, TIMP3, and TIMP4, which have different tissue-specific expression patterns and substrate specificities [16]. They can act either directly through cell surface receptors or indirectly through modulation of protease activity to modulate cell functions. TIMPs have been shown to be regulated by histone modification enzymes, such as EZH2 and TET1, in cancer [17, 18]. However, it is not clear whether KDM1A can also regulate specific TIMP genes and thereby promote cancer metastasis.

In metastasized tumors, Jun N-terminal kinase (JNK) phosphorylation has been found to be enhanced [19]. Higher levels of JNK phosphorylation are correlated with more advanced stages of cancer [20, 21]. JNK regulates cell migration by promoting the tyrosine phosphorylation of paxillin [22]. The mechanism of epigenetic regulation by which the JNK signaling pathway acts has yet to be defined.

Here we demonstrate that KDM1A advances tumor metastasis by epigenetically silencing the TIMP3 transcription, which in turn stimulates MMP2 expression and JNK phosphorylation. Our work reveals a new mechanism for how KDM1A promotes metastasis in NSCLC, and suggests KDM1A may be a potential therapeutic target for treating NSCLC.

## RESULTS

### KDM1A expression is elevated in NSCLC patients

**Figure 1:**
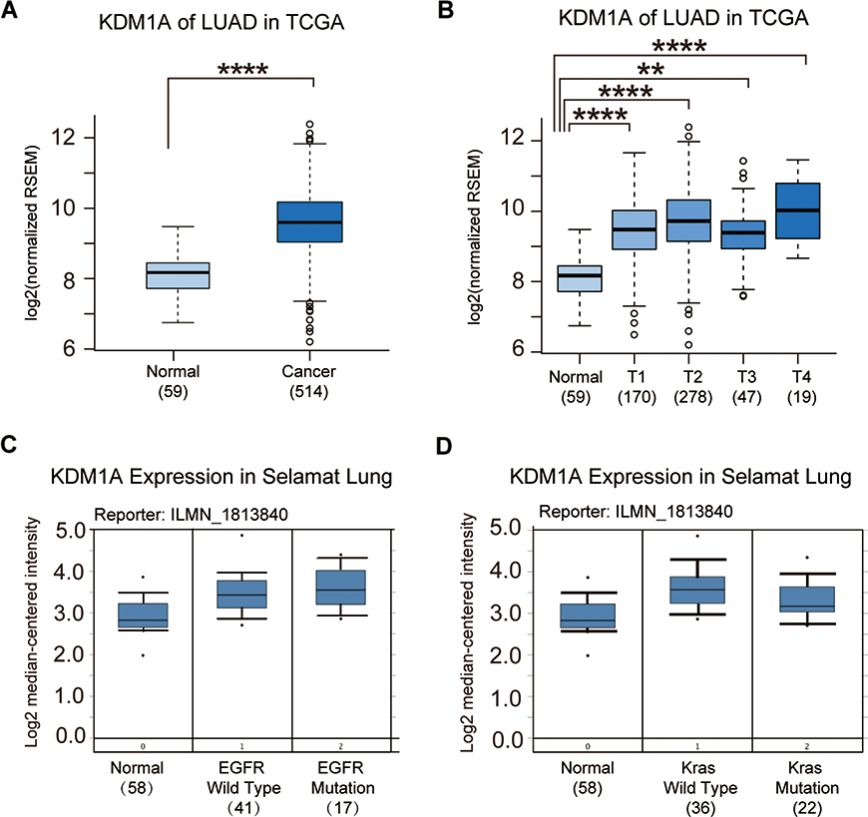
KDM1A is significantly up-regulated in NSCLC (**A**) KDM1A expression in normal lung tissue (Normal) and NSCLC (Cancer) (data from lung Adenocarcinoma (LUAD) of the TCGA database). The log2 fold change and *p* value of KDM1A expression are: 0.68 (*p* = 5.01E-5, Cancer vs. Normal). (**B**) KDM1A expression in normal lung tissue (Normal) and all stages (T1, T2, T3, and T4) of LUAD (data from the TCGA database). Log2 fold changes and *p* values of KDM1A expression are: 0.58 (*p* = 0.00036, T1 vs. Normal), 0.75 (*p* = 1.12E-5, T2 vs. Normal), 0.57 (*p* = 0.003, T3 vs. Normal), and 0.95 (*p* = 0.00012, T4 vs. Normal). (**C**) KDM1A expression in normal lung tissue and NSCLC patients with either a wild type or mutant EGFR gene in the Selamat Lung dataset (data from the Oncomine database). (**D**) KDM1A expression in normal lung tissue and NSCLC patients with either a wild type or mutant Kras gene in the Selamat Lung dataset (data from the Oncomine database). Reporter stands for the probe name used in the experiments. The number in the parenthesis represents the sample size.

To examine the expression pattern of KDM1A in NSCLC patients, we analyzed the gene expression data from lung adenocarcinoma in the TCGA database. We found that KDM1A was not only up-regulated in tumors compared to non-tumor controls but also significantly up-regulated in all stages of NSCLC, from stage 1 (T1) to stage 4 (T4) (Figure 1A and 1B). Because EGFR and Kras mutations are two well-known oncogenic driver mutations in NSCLC patients, using the Oncomine database, we examined the KDM1A expression pattern based on EGFR and Kras mutation status in NSCLC, and found that regardless of mutation status, KDM1A was up-regulated in all NSCLCs. However, patients with EGFR mutations showed higher KDM1A expression than those that were EGFR wild type, whereas patients with Kras mutations showed lower KDM1A expression than those that were Kras wild type (Figure 1C and 1D).

### KDM1A promotes tumor growth and metastasis in NSCLC

**Figure 2:**
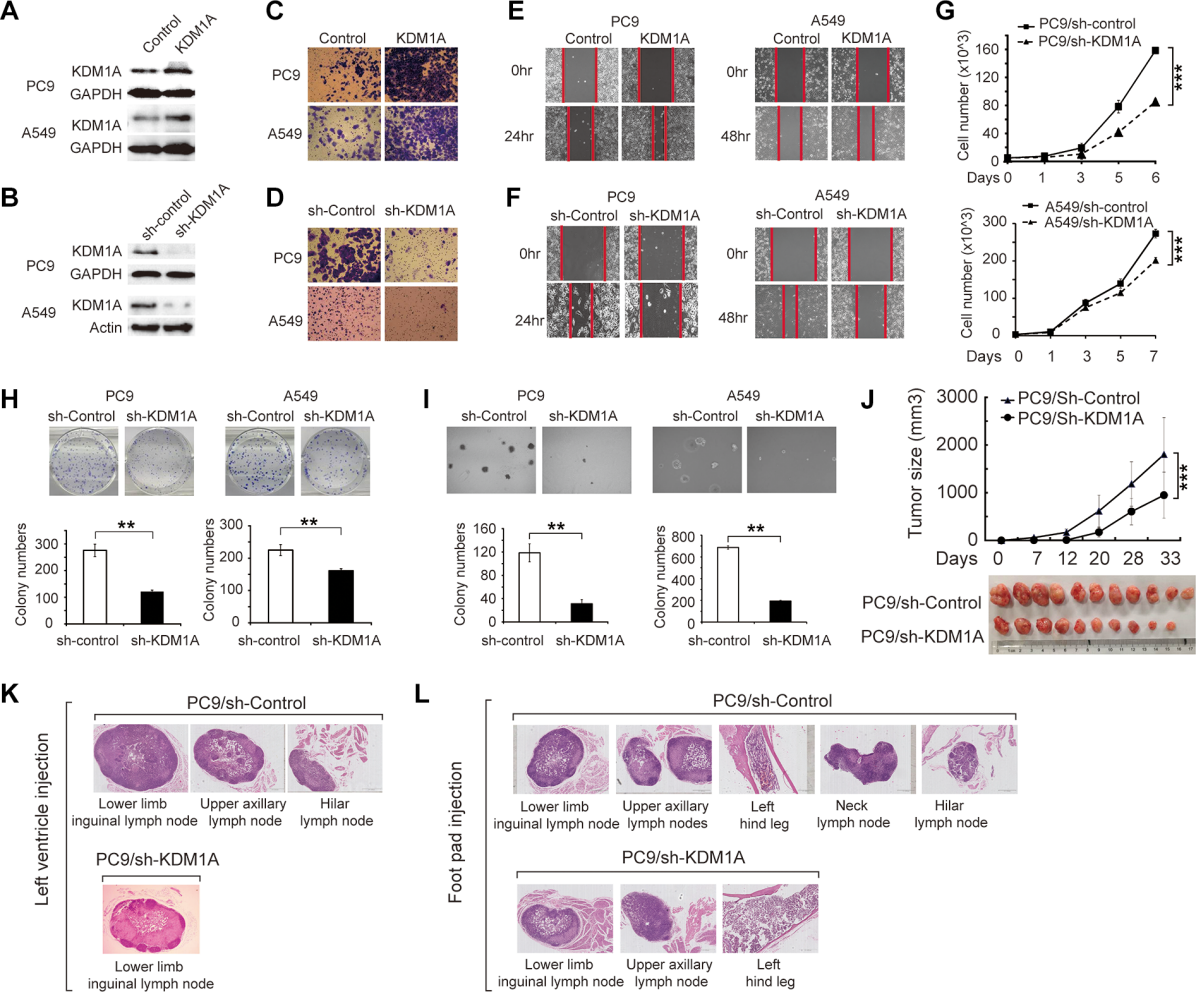
KDM1A promotes tumor growth and metastasis in NSCLC (**A**, **B**) Validation of KDM1A overexpression (A) and knockdown (B) in PC9 and A549 cells by WB. GAPDH serves as a loading control. (**C**) Invasion capacities of PC9 or A549 cells transfected with either the control plasmid (Control) or KDM1A overexpression plasmid (KDM1A). (**D**) Invasion capacities of PC9 or A549 cells stably expressing the control shRNA (sh-Control) or KDM1A shRNA (sh-KDM1A). (**E**) Migration capacities of PC9 or A549 cells transfected with either the control plasmid (Control) or KDM1A overexpression plasmid (KDM1A). (**F**) Migration capacities of PC9 or A549 cells stably expressing the control shRNA (sh-Control) or KDM1A shRNA (sh-KDM1A). The red line indicates the edge of migrating cells at a given time point. (**G**, **H**, **I**) PC9 or A549 cells stably expressing the control shRNA (sh-Control) or KDM1A shRNA (sh-KDM1A) were subjected to: cell proliferation assay at different time points (0, 1, 3, 5, 6 or 7 days) (G), colony formation assay (H), or anchorage-independent colony formation assay in soft agar (I). Bar graphs show the quantification of colony number in each condition. (**J**) Growth curve of xenograft tumors derived from PC9 cells stably expressing the control shRNA (sh-Control) or KDM1A shRNA (sh-KDM1A) (upper panel). After approximately 5 weeks, xenograft tumors were dissected and photographed (lower panel). Each group contained 10 or 11 mice. (**K**, **L**) H&E-stained images of metastatic nodes in the mice left ventricle injection and foot pad injection experiments. (K) For the left ventricle injection experiments: in the mice injected with PC9 cells stably expressing the control shRNA (PC9/sh-Control), metastatic nodes were found in the lower inguinal, upper axillary, and hilar lymph nodes, but in the mice injected with PC9 cells stably expressing KDM1A shRNA (PC9/sh-KDM1A), metastatic nodes were found only in the lower inguinal and not in other lymph nodes. (L) For the foot pad injection experiments: in the mice with PC9/sh-Control, metastatic nodes were found in the lower inguinal, upper axillary, neck, and hilar lymph nodes, as well as in the left hind leg; but in the mice with PC9/sh-KDM1A, metastatic nodes were found only in the lower inguinal and upper axillary lymph nodes, and in the left hind leg, but not in other parts.

To study biological consequences of KDM1A up-regulation in NSCLC cells with different oncogenic driver mutations, we examined KDM1A expression in multiple NSCLC cell lines, including PC9, PC9R, H1650, H292, H1975, 95D, HCC827, and A549 cells, and found that KDM1A expression varied considerably among these cells. We chose PC9 cells with an EGFR-activating mutation (exon19: delE746-A750) and A549 cells with a Kras mutation (exon 2: G12S) for the subsequent study (Supplementary Figure S1A). Our data revealed that overexpressing KDM1A enhanced cell invasion and migration in both types of cells (Figure 2A, 2C, and 2E). Conversely, in cells stably expressing KDM1A shRNA, KDM1A expression was greatly reduced (Figure 2B), and invasion and migration capacities of these cells were also decreased compared to those in cells expressing the control shRNA (Figure 2D, 2F). We got similar phenotypes when using HCC827, another NSCLC cell line carrying the same EGFR activating mutation as in PC9 cells (Supplementary Figure S1B). Next, cells exhibiting stable KDM1A knockdown showed reduced cell proliferation (Figure 2G), decreased colony formation in the culture dish (Figure 2H), and decreased anchorage independent colony formation in soft agar (Figure 2I). In addition, KDM1A knockdown delayed cell cycle progression by increasing the length of the G1 phase and decreasing the length of the S phase (Supplementary Figure S1C and S1D), but had no significant effect on apoptosis: no cleaved caspase 3 or PARP bands were detected by western blotting (WB), as compared to those in cells expressing the control shRNA (Supplementary Figure S1E). We did not use a FITC Annexin V apoptosis detection kit to assay apoptosis because PC9 cells stably expressing KDM1A shRNA contain a fluorescent dye that interferes with the fluorescence intensity read-out of FITC Annexin V. Finally, to study whether EGFR inhibition has any effect on KDM1A expression, we treated both PC9 and A549 cells with 0, 1, or 3 μm of gefitinib, an EGFR tyrosine kinase inhibitor, for 2 days, and found that KDM1A expression was not changed significantly, suggesting short-term inhibition of EGFR kinase activity alone has no substantial effect on KDM1A expression (Supplementary Figure S1F).

To test the effect of KDM1A knockdown on tumorigenesis *in vivo*, we performed xenograft experiments in nude mice. Cells stably expressing KDM1A shRNA or the control shRNA were injected subcutaneously into the right axillary region of nude mice, and the sizes of xenograft tumors were measured every week. Five weeks later, tumors were isolated and imaged. We found that xenograft tumors derived from PC9 cells that stably expressed KDM1A shRNA exhibited significantly slower growth rates and smaller tumor sizes, on average, compared to cells expressing the control shRNA (Figure 2J).

To further investigate the effect of KDM1A knockdown on tumor metastasis *in vivo*, we established two models using nude mice. First, cells were injected into the left ventricles of the mice, which allowed them to metastasize into different parts of the body. The metastatic capacity of these cells can be assessed by determining the number of metastasized organs. Four weeks after injection, PC9 cells stably expressing KDM1A shRNA had metastasized only into the lower limb inguinal lymph nodes, whereas PC9 cells stably expressing the control shRNA had metastasized into more lymph nodes, including the lower limb inguinal, upper axillary, and hilar lymph nodes (Figure 2K).

In the second model examining tumor metastasis *in vivo*, cells were injected into the left hind limb footpads of nude mice. Several weeks after injection, PC9 cells stably expressing KDM1A shRNA had metastasized only into the lower limb inguinal and upper axillary lymph nodes, as well as into the left hind leg bone, whereas PC9 cells expressing the control shRNA had metastasized into more parts of the body, including the lower limb inguinal, upper axillary, neck, and hilar lymph nodes, as well as the left hind leg bone (Figure 2L). Together, these results indicate that KDM1A promotes tumorigenesis and metastasis of NSCLC cells both *in vitro* and *in vivo*.

### TIMP3 expression is repressed by KDM1A

**Figure 3:**
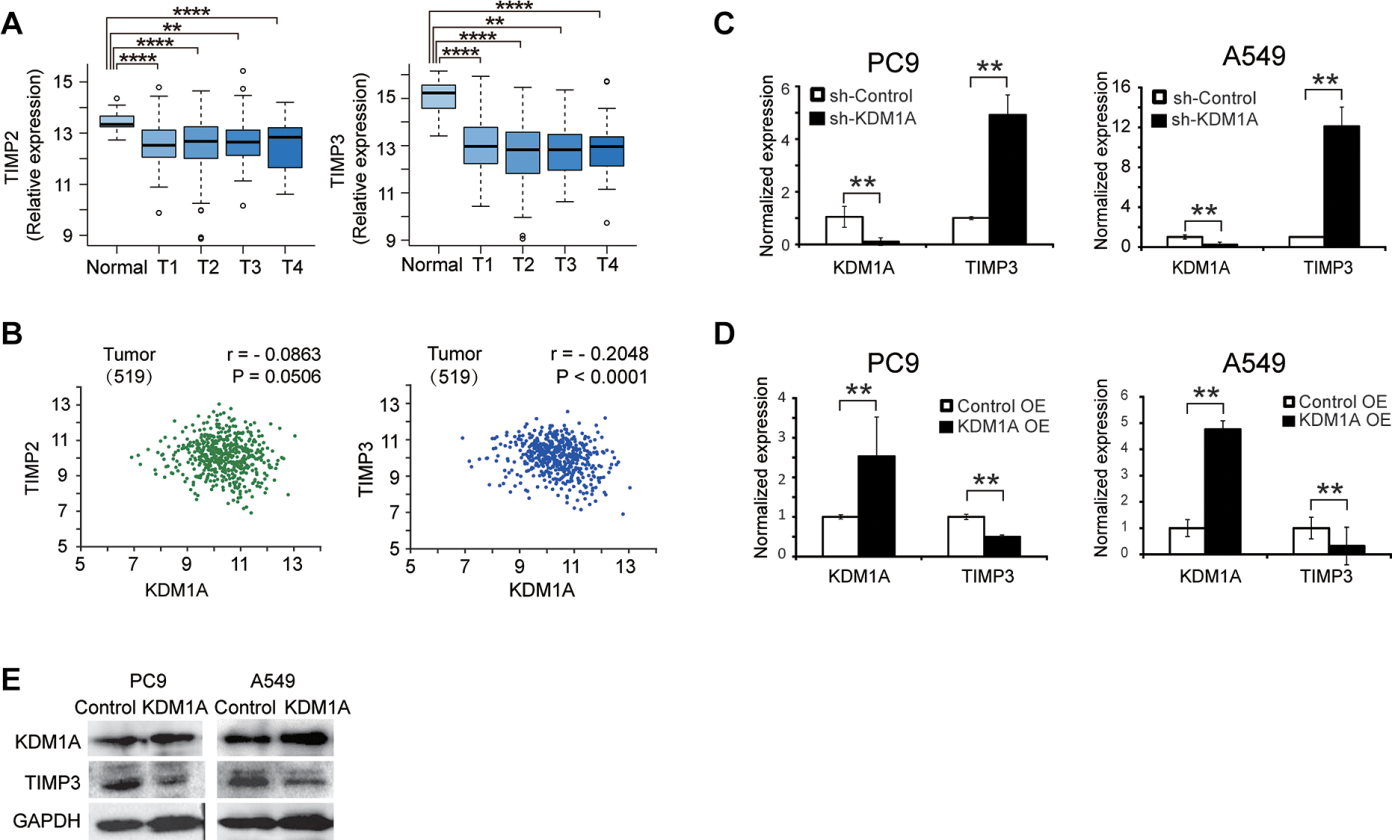
TIMP3 expression is negatively correlated with KDM1A in NSCLC, and is repressed by KDM1A (**A**) Comparison of the relative expression of TIMP2 and TIMP3 in different tumor stages (T1, T2, T3, and T4) of NSCLC patients compared to non-tumor control (normal) (data from LUAD of the TCGA database). Log2 fold changes in TIMP2 expression are: −0.78 (*p* = 6.87E-10, T1 vs. Normal), −0.67 (*p* = 1.98E-6, T2 vs. Normal), −0.44 (*p* = 0.0076, T3 vs. Normal), and −0.56 (*p* = 0.024, T4 vs. Normal). Log2 fold changes in TIMP3 expression are: −1.88 (*p* = 5.43E-42, T1 vs. Normal), −2.02 (*p* = 2.89E-47, T2 vs. Normal), −2.06 (*p* = 1.26E-28, T3 vs. Normal), and −1.53 (*p* = 1.56E-10, T4 vs. Normal). (**B**) Correlation analysis between KDM1A and TIMP2 expressions (left panel), or between KDM1A and TIMP3 expressions (right panel) in tumors (data from LUAD of the TCGA database). Correlation coefficient (r) and *P* value (P) are calculated. Numbers within the parentheses represent the sample size. (C, D) Normalized KDM1A and TIMP3 expressions in PC9 or A549 cells expressing the control shRNA (sh-Control) or KDM1A shRNA (sh-KDM1A) (**C**), or PC9 or A549 cells transfected with either the control (Control OE) or KDM1A overexpression plasmid (KDM1A OE) (**D**), detected by real-time RT-PCR. Each condition was performed in triplicates. Data are represented as mean (SD). (**E**) WB detection of KDM1A and TIMP3 expressions in PC9 or A549 cells transfected with either the control (Control) or KDM1A overexpression plasmid (KDM1A). GAPDH serves as a loading control.

Because the TIMP family of proteins, including TIMP1, TIMP2, TIMP3, and TIMP4, are important regulators of cell migration and invasion in the extracellular matrix, we postulated that they may be key target genes of KDM1A. First, we analyzed the expression of TIMPs in lung adenocarcinoma in the TCGA database and found that only TIMP2 and TIMP3 expression were significantly down-regulated in tumors compared to normal controls (Figure 3A). TIMP1 was significantly up-regulated in NSCLC, and TIMP4 expression was not significantly changed (data not shown). In NSCLC, KDM1A expression was more significantly negatively correlated with TIMP3 expression (*r* = −0.2048, *P* < 0.0001), than with the other TIMPs: TIMP2 (*r* = −0.0791, *P* = 0.0506) (Figure 3B), TIMP1 (*r* = −0.1285, *P* = 0.0034), and TIMP4 (*r* = −0.11, *P* = 0.0724) (TIMP1 and TIMP4 are not shown). In addition, we also analyzed the correlation coefficients between KDM1A and TIMP3 expressions in normal tissue and different tumor stages in lung adenocarcinoma in the TCGA database, and found that KDM1A expression is correlated with TIMP3 expression more significantly in T1 (*r* = −0.2423, *P* = 0.0015) and T2 stages (*r* = −0.2108, *P* = 0.0004) than in T3 and T4 stages. Due to smaller sample sizes in T3 and T4 stages, their correlation coefficient estimation may be less accurate than those in T1 and T2 stages (Supplementary Figure S2A to S2E). Finally, we observed that, clinical NSCLC samples carrying EGFR mutations contain higher KDM1A level and lower TIMP3 expression, whereas those carrying Kras mutations have lower KDM1A level and higher TIMP3 expression (Supplementary Figure S2F). These analyses indicate that TIMP3 may be more likely to be a target gene that is repressed by KDM1A than other TIMPs.

We next sought to confirm whether KDM1A represses TIMP3 expression in NSCLC cells. First, we found that, in PC9 and A549 cells stably expressing KDM1A shRNA, TIMP3 RNA was significantly up-regulated compared to the cells expressing the control shRNA (Figure 3C). Similarly, transient knockdown of KDM1A by siRNA also reactivated TIMP3 expression in HCC827 cells (Supplementary Figure S1G). Conversely, when KDM1A was overexpressed in PC9 and A549 cells, the TIMP3 RNA level was significantly decreased (Figure 3D), as was its protein level (Figure 3E).

To determine whether KDM1A knockdown activates TIMP3 *in vivo*, we performed gene expression microarray analyses using xenograft tissues derived from PC9 cells stably expressing either KDM1A shRNA or the control shRNA. Microarray analysis identified 2994 genes encoding mRNAs and noncoding RNAs that were differentially expressed (*P* < 0.05) between the two groups (Supplementary Table S1A). Among them, 1078 are protein-coding genes, as shown in a clustering heatmap (Supplementary Figure S3A). TIMP3 is one gene that was significantly up-regulated (*P* < 0.05) (Supplementary Table S1A and S1B). Furthermore, gene ontology (GO) analysis revealed that, in xenograft tissues derived from PC9 cells stably expressing KDM1A shRNA, the top biological process enriched among up-regulated genes was the cell surface-linked signal transduction pathway (Supplementary Figure S3B and Supplementary Table S1C), which is consistent with the role of TIMP3 in mediating cell surface receptor activity and signaling. The top biological process enriched among down-regulated genes was the cell cycle (Supplementary Figure S3B and Supplementary Table S1D), which is in line with our previous observation that KDM1A knockdown inhibited cell cycle progression. Therefore, our data suggested that KDM1A represses TIMP3 gene expression in NSCLC cells *in vitro* and *in vivo*.

### TIMP3 suppresses KDM1A-mediated cell invasion and migration

**Figure 4:**
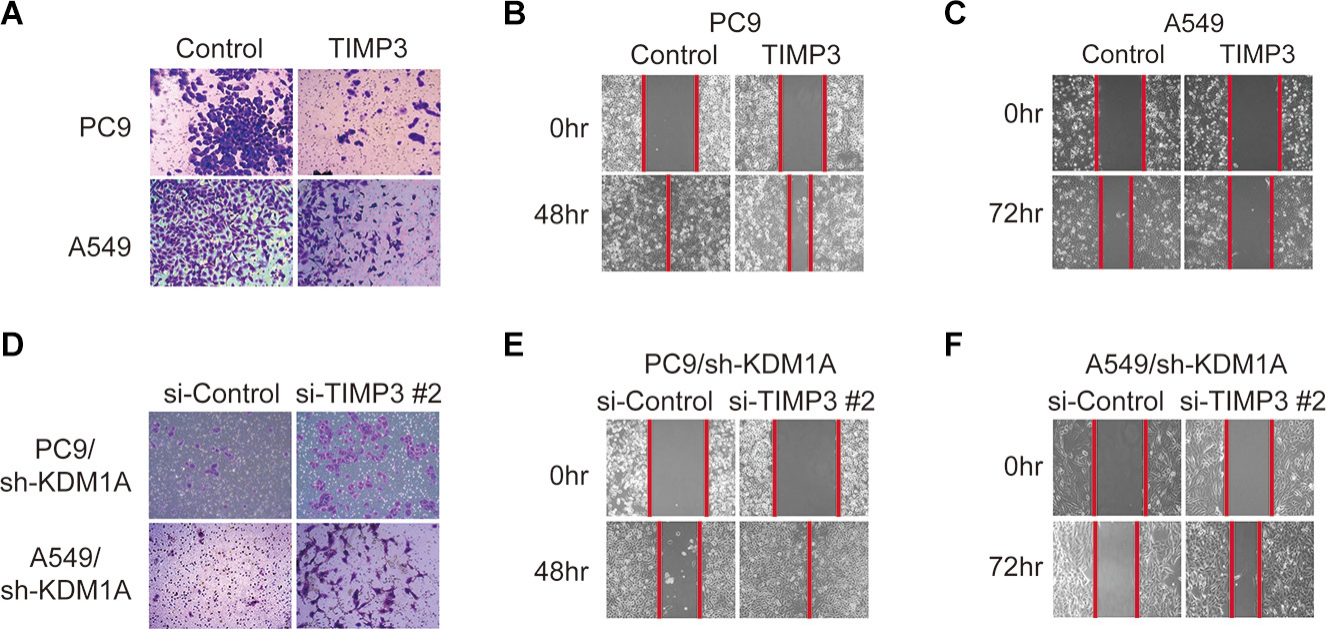
TIMP3 suppresses KDM1A-mediated cell invasion and migration (**A**, **B**, **C**) PC9 or A549 cells transfected with either the control plasmid (Control) or TIMP3 overexpression plasmid (TIMP3) were subjected to cell invasion assay (A) and cell migration assays (B, C). (**D–F**) PC9 or A549 cells stably expressing KDM1A shRNA (sh-KDM1A) were transfected with either the control siRNA (si-Control) or TIMP3 siRNA (si-TIMP3 #2), and subjected to cell invasion assay (D) and cell migration assays (E, F). The red line indicates the edge of migrating cells at a given time point.

To study whether repression of TIMP3 gene expression is necessary for KDM1A-induced cell invasion and migration in PC9 and A549 cells, we overexpressed TIMP3 in these cells and found that the capacity of these cells to invade and migrate was significantly reduced and that the inhibition of migration was stronger in PC9 than in A549 cells (Figure 4A and 4C). Similar results were obtained in HCC827 cells (Supplementary Figure S1H). Conversely, when TIMP3 was knocked down by siRNA in PC9 and A549 cells that stably expressed KDM1A shRNA, the capacity of these cells to invade and migrate was significantly rescued in both cells lines, and the rescuing effect on migration was more obvious in PC9 than in A549 cells (Figure 4D to 4F), indicating that PC9 cells are more sensitive to KDM1A-mediated effects. Overall, we conclude that KDM1A promotes cell invasion and migration at least in part by repressing TIMP3 expression.

### KDM1A demethylates H3K4me2 at the TIMP3 promoter

**Figure 5:**
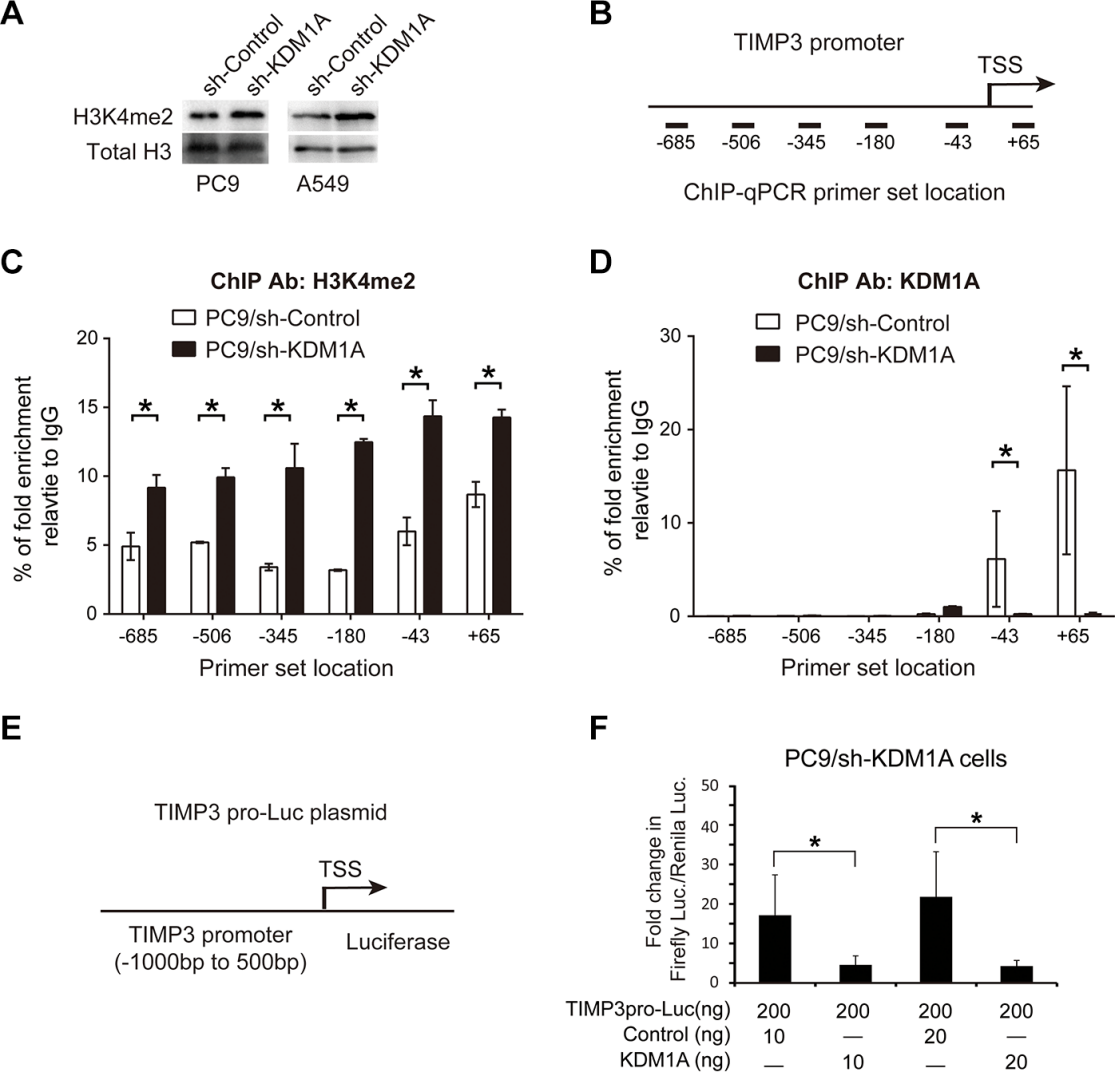
KDM1A removes H3K4me2 at the TIMP3 promoter and represses its promoter activity (**A**) WB detection of the H3K4me2 level in PC9 and A549 cells expressing the control shRNA (sh-Control) or KDM1A shRNA (sh-KDM1A). Total histone H3 serves as a loading control. (**B**) Schematic drawing of the TIMP3 promoter region and ChIP-qPCR primer set locations (−685, −506, −345, −180, −43, and +65), relative to the TSS (transcriptional start site). TSS is assigned as the “+1” position. (C, D) ChIP-qPCR was performed in PC9 cells expressing the control shRNA (sh-Control) or KDM1A shRNA (sh-KDM1A) using an anti-H3K4me2 antibody (**C**) or an anti-KDM1A antibody (**D**). Bar graphs show percentages of fold enrichment of H3K4me2 (C) or KDM1A (D) at different locations of the TIMP3 promoter region, relative to IgG immunoprecipitation. (**E**) Schematic drawing of the TIMP3 promoter luciferase reporter construct (TIMP3pro-Luc plasmid). TIMP3 promoter region spanning from −1000 bp to 500 bp with respect to the TSS was cloned into luciferase reporter vector (pGL3). (**F**) TIMP3pro-Luc plasmid was co-transfected with either KDM1A overexpression plasmid (KDM1A) or the control plasmid (Control) into PC9 cells stably expressing KDM1A shRNA, and relative luciferase activity was measured 72 hours later. Each condition was performed in six replicates. Data are represented as mean (SD).

KDM1A is a histone modification enzyme that demethylates H3K4me2 at its target gene promoter and recruits the repressive chromatin complex to silence gene expression [5]. In both PC9 and A549 cells stably expressing KDM1A shRNA, we found that the global H3K4me2 level was up-regulated considerably upon KDM1A depletion (Figure 5A). To investigate whether KDM1A silences TIMP3 expression by demethylating H3K4me2 at its promoter, we performed ChIP-qPCR using antibodies against KDM1A and the histone mark H3K4me2, and found that, in PC9 cells with stable KDM1A knockdown, H3K4me2 enrichment was significantly increased at the TIMP3 promoter region and its upstream region as well (Figure 5B and 5C), whereas KDM1A enrichment was dramatically reduced around the TIMP3 promoter region, compared to those in the control cells (Figure 5D). Interestingly, KDM1A seems to bind specifically to the TIMP3 promoter region (−43 to +65, relative to the transcriptional start site (TSS)), but not to its upstream region (−685 to −180, relative to the TSS) (Figure 5D).

To further examine whether KDM1A could directly repress TIMP3 promoter activity, we cloned a DNA fragment containing 1.5 kb of the upstream region of the TIMP3 promoter, which has been previously well-characterized [23], into the pGL3 basic luciferase plasmid and conducted luciferase reporter assays in the presence or absence of KDM1A overexpression plasmid or the control plasmid. We found that KDM1A overexpression specifically repressed the TIMP3 promoter activity and that this effect was dose-dependent (Figure 5E and 5F), which indicates that this upstream region may contain cis-elements that can recruit KDM1A to the TIMP3 promoter. In conclusion, these findings demonstrate that KDM1A silences the TIMP3 transcription by targeting its promoter and removing H3K4me2 from its promoter.

### KDM1A enhances JNK phosphorylation by repressing TIMP3 and increasing MMP2 expression

**Figure 6:**
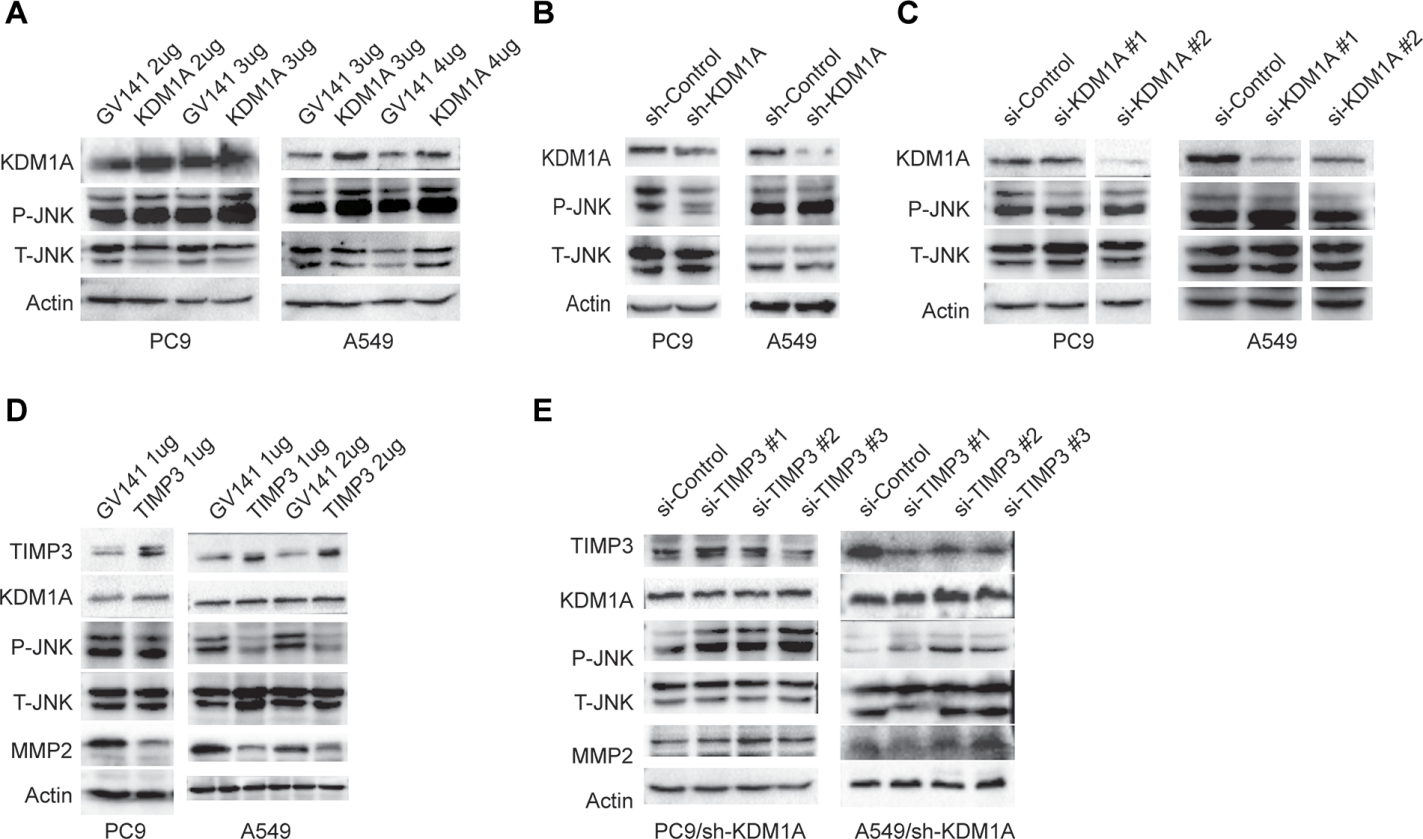
JNK phosphorylation is enhanced by KDM1A overexpression, but repressed by TIMP3 overexpression (**A**) PC9 or A549 cells transfected with either the control (GV141) or KDM1A overexpression plasmid (KDM1A) at given amounts (2, 3, or 4 μg) were subjected to WB, to detect the following protein expressions: KDM1A, total and phosphorylated JNK (T- and P-JNK). (**B**, **C**) PC9 or A549 cells stably expressing the control shRNA (sh-Control) or KDM1A shRNA (sh-KDM1A) (B), or PC9 or A549 cells transiently transfected with the control siRNA (si-Control) or two different KDM1A siRNA (si-KDM1A #1, #2) (C), were subjected to WB, to detect the following protein expressions: KDM1A, total and phosphorylated JNK. Images of WB detection for KDM1A and Actin in A549 cells (sh-Control and sh-KDM1A) was also shown in Figure 2B. (**D**) PC9 or A549 cells transfected with either the control (GV141) or TIMP3 overexpression plasmid (TIMP3) at given amounts (1, or 2 μg) were subjected to WB, to detect the following protein expressions: TIMP3, KDM1A, T- and P-JNK, and MMP2. (**E**) PC9 or A549 cells stably expressing the control shRNA (sh-Control) or KDM1A shRNA (sh-KDM1A) were transfected with either the control siRNA (si-Control) or three different TIMP3 siRNA (si-TIMP3 #1, #2, #3), were subjected to WB, to detect the following protein expression: TIMP3, KDM1A, T- and P-JNK, and MMP2. Actin serves as a loading control.

JNK is an important kinase that is involved in regulating cell polarity and migration. We next sought to determine whether KDM1A-mediated cell invasion and migration involves JNK activation. First, we overexpressed KDM1A in both PC9 and A549 cells and found that JNK phosphorylation (P-JNK), but not the total level of JNK (T-JNK), was enhanced in these cells (Figure 6A). Conversely, KDM1A knockdown by shRNA or two different siRNAs resulted in reduced JNK phosphorylation, but no reduction in the total level of JNK in these cells (Figure 6B and 6C). Next, to examine whether TIMP3 can repress JNK phosphorylation, we overexpressed TIMP3 in both PC9 and A549 cells and found that JNK phosphorylation, but not the total level of JNK, was decreased significantly, and that the level of MMP2 protein was also decreased, upon TIMP3 overexpression. However, it has no effect on KDM1A expression (Figure 6D). Conversely, TIMP3 knockdown (by siRNA) in cells stably expressing KDM1A shRNA restored JNK phosphorylation and also increased the MMP2 protein level (Figure 6E). Therefore, these results suggest that KDM1A promotes JNK phosphorylation and MMP2 expression by repressing TIMP3, which is a downstream target for KDM1A.

### Pharmacological inhibition of KDM1A inhibits cell proliferation, invasion, and migration in NSCLC cells

**Figure 7:**
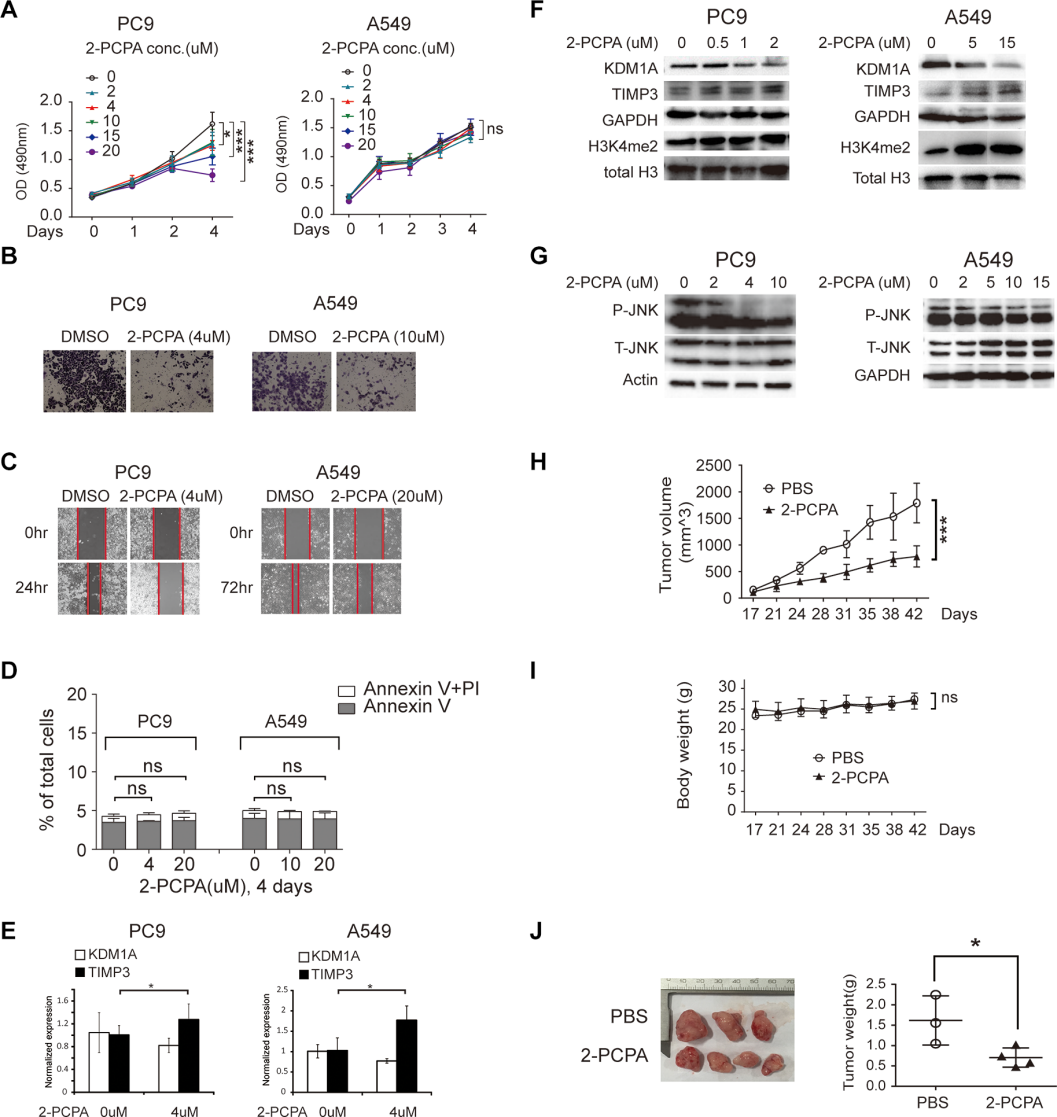
2-PCPA treatment inhibits cell proliferation, invasion, and migration in NSCLC cells (**A**) PC9 and A549 cells were treated with 2-PCPA at various concentrations (0, 2, 4, 10, 15, 20 μM), and subjected for cell proliferation assay, at given time points (0, 1, 2, 3, or 4 days). (B, C) PC9 and A549 cells were treated with either DMSO or 2-PCPA (4, 10, or 20 μM), and subjected to cell invasion assay (**B**), and cell migration assays (**C**). The red line indicates the edge of migrating cells at a given time point. (**D**) PC9 and A549 cells were treated with either DMSO or 2-PCPA (4, 10, or 20 μM), and subjected to cell apoptosis assay using BD Annexin V FITC kit. The bar graph represents percentages of cells expressing early (Annexin V) or late (PI) apoptosis markers. (**E**) Normalized KDM1A and TIMP3 expressions in PC9 and A549 cells treated with 0 or 4 μM 2-PCPA for 2 days, detected by real time RT-PCR. Each condition was performed in triplicates. Data are represented as mean (SD). (F-G) PC9 and A549 cells were treated with 2-PCPA at various concentrations (0, 0.5, 1, 2, 5, and 15 μM (**F**), and 0, 2, 4, 5, 10, and 15 μM (**G**), and subjected to WB, to detect the following protein expressions: KDM1A, TIMP3, H3K4me2 (F), P-JNK, and T-JNK (G). GAPDH, Actin, and total histone H3 serve as loading controls. (**H**, **I**,) Growth curve of PC9 cells derived xenograft tumors (H) and mice body weight (I) with either PBS (control) or 2-PCPA treatment. (**J**) Image of dissected xenograft tumors (left panel). Tumor weight comparison of two treatment groups by *t* test (right panel).

2-PCPA is an irreversible inhibitor of the enzyme monoamine oxidase (MAO) [24] that has been also used as a small molecular inhibitor of KDM1A in many studies [25]. To determine whether it could inhibit KDM1A-mediated phenotypes in NSCLC cells, we treated both PC9 and A549 cells with different concentrations of 2-PCPA (0, 2, 4, 10, 15, and 20 μM), which is comparable with the concentrations of 2-PCPA used in other cancer studies [25, 26], and found that it inhibited cell proliferation, invasion, and migration in both PC9 and A549 cells (Figure 7A, 7B, and 7C). Interestingly, 2-PCPA had a more potent inhibitory effect in PC9 than in A549 cells. For example, 4 μM 2-PCPA significantly inhibited cell proliferation, invasion, migration in PC9 cells, but only 20 μM 2-PCPA had a similar effect on A549 cells. To determine whether above changes induced by 2-PCPA treatment was due to increased cell apoptosis, we treated both cells with different concentrations of 2-PCPA (0, 4, and 20 μM for PC9, 0, 10, and 20 μM for A549) for four days, and found that apoptosis rates remained approximately the same for all treatment conditions (Figure 7D). In addition, 2-PCPA treatment led to the up-regulation of TIMP3 and the slight down-regulation of KDM1A at the RNA level (Figure 7E). Similarly, by WB analysis, we found that TIMP3 expression was increased, but KDM1A expression was decreased, upon treatment with 2-PCPA. Interestingly, the total level of H3K4me2 in these cells was increased, indicating that 2-PCPA inhibited the H3K4me2 demethylation activity of KDM1A (Figure 7F). In addition, 2-PCPA treatment also significantly inhibited JNK phosphorylation at only 4 μM in PC9 cells but required 15 μM in A549 cells (Figure 7G).

To study whether 2-PCPA inhibits tumorigenesis *in vivo*, we treated mice carrying PC9 cells derived xenograft tumors with PBS or 2-PCPA by intragastric injection, using a similar dosage of 2-PCPA as described in other studies [27], our results demonstrate that 2-PCPA can effectively suppress tumorigenesis *in vivo* (Figure 7H–7J). Finally, we found 2-PCPA treatment had no effect on MAO-A/B expression in these cells (Supplementary Figure S1I), indicating these changes were mediated by KDM1A inhibition. Overall, these observations are consistent with those from our study using a stable KDM1A knockdown by shRNA (Figure 2A–2L). In addition, our results suggest that certain types of NSCLC cells exhibiting high levels of KDM1A expression, such as PC9, may be more sensitive to KDM1A inhibition.

### High expression of KDM1A and low expression of TIMP3 are significantly associated with a poor survival in NSCLC patients

**Figure 8:**
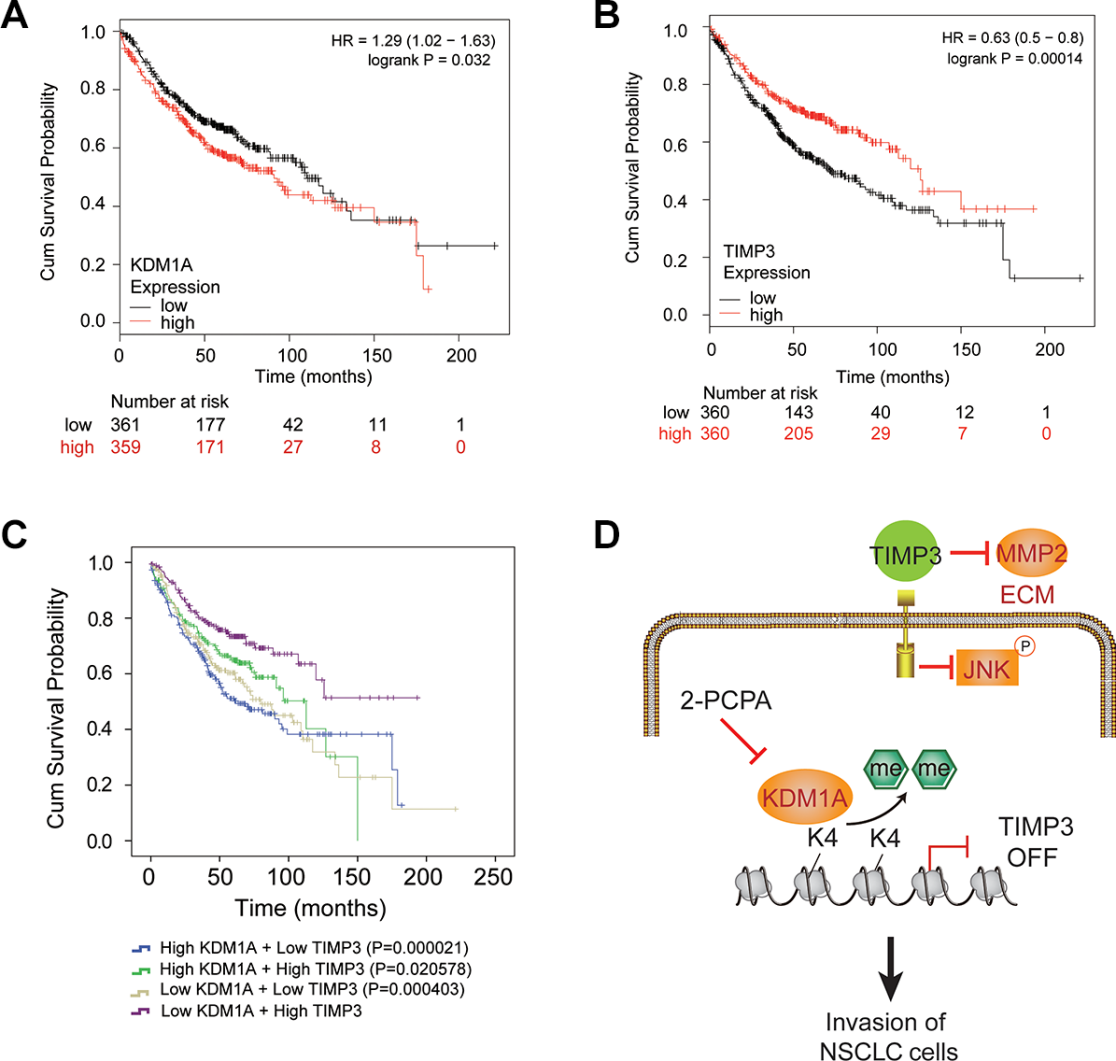
Kaplan-Meier curve analysis of overall survival probabilities of NSCLC patients based on KDM1A and TIMP3 expressions (**A**) High KDM1A expression is associated with a poor prognosis in NSCLC patients, compared to those with low KDM1A expression (*P* = 0.032). Numbers at risk for each time point were listed below time points. (**B**) Low TIMP3 expression is associated with a poor prognosis in NSCLC patients, compared to those with high TIMP3 expression (*P* = 0.00014). (**C**) NSCLC patients were divided into four groups based on KDM1A and TIMP3 expressions. The comparison of survival probabilities among these groups was done by the logrank (Mantel-Cox) test. *P* values are given when using the group with low KDM1A and high TIMP3 expressions as the reference group. (**D**) Model of KDM1A's role in promoting metastasis of NSCLC. KDM1A silences TIMP3 expression by removing H3K4me2 in its promoter, thus releasing TIMP3′s inhibition on MMP2 expression and JNK phosphorylation. This leads to enhanced cell migration and invasion of NSCLC cells. Small molecular inhibitors of KDM1A, such as 2-PCPA, may effectively inhibit the metastasis of certain types of NSCLC.

To study whether high KDM1A expression or low TIMP3 expression is correlated with poor survival in NSCLC patients, we carried out survival analyses based on KDM1A or TIMP3 expression in NSCLC patients from a released database (http://kmplot.com/) [28] (Supplementary Table S2A and S2B). We found that high KDM1A or low TIMP3 expression was significantly associated with poor prognosis of NSCLC patients, irrespective of grade and histology, with logrank (Mantel-Cox) *P* values of 0.032 for KDM1A and 0.00014 for TIMP3 (Figure 8A and 8B). To further study the impact of expressions of both genes on patient survival, we divided these patients into four groups: high KDM1A and low TIMP3 expressions, high KDM1A and high TIMP3 expressions, low KDM1A and low TIMP3 expressions, low KDM1A and high TIMP3 expressions. Using a logrank test, we found that NSCLC patients with high KDM1A and low TIMP3 expressions are more significantly associated with poor prognosis (*P* = 0.000021), than other groups of patients with high KDM1A and high TIMP3 (*P* = 0.020578), or low KDM1A and low TIMP3 expressions (*P* = 0.000403), when compared to those with low KDM1A and high TIMP3 expressions (Figure 8C). *P* values were calculated for the pair-wise comparison among four groups (Supplementary Table S2C). Therefore, our analysis suggests that high KDM1A and low TIMP3 expressions may promote tumor metastasis, which is associated with poor NSCLC prognosis.

## DISCUSSION

In the current study, we demonstrate that KDM1A promotes tumor metastasis both *in vitro* and *in vivo*, and its higher expression is significantly correlated with poor prognosis in NSCLC patients. Importantly, KDM1A exerts these effects by epigenetically silencing TIMP3 transcription, which in turn increases MMP2 expression in the extracellular matrix (ECM) and JNK phosphorylation in the cytoplasm of tumor cells, thus contributing to cell invasion and migration in NSCLC (Figure 8D).

KDM1A may represent an important therapeutic target for NSCLC and other cancers [29–31]. An earlier study has shown that the pharmacological inhibition of KDM1A reduces tumor growth and reactivates the all-*trans*-retinoic acid differentiation pathway in acute myeloid leukemia (AML) [32]. Clinical trials for AML and small cell lung carcinoma using the KDM1A inhibitor GSK2879552 are underway (http://clinicaltrials.gov). In our study, we found that a treatment with a KDM1A inhibitor (2-PCPA) reduced tumor cell proliferation, migration, and invasion. Specifically, PC9 cells with an EGFR mutation were more sensitive to 2-PCPA treatment than A549 cells with a Kras mutation. We speculate that may be due to higher KDM1A expression in PC9 cells than in A549 cells. Interestingly, NSCLCs carrying EGFR mutations have higher KDM1A expression than those carrying Kras mutations, suggesting that KDM1A inhibition may be more effective in NSCLC cells with EGFR mutations than in those with Kras mutations. Further investigation of this differential response may contribute to more effective treatment of NSCLC. Finally, our study also suggests that overexpression of TIMP3 may be used to treat NSCLC. For example, adenovirally transferred TIMP3 reduces adhesion, migration, and invasion behaviors in colorectal cancer cells and suppressed tumor growth *in vivo* [33].

The mechanism by which KDM1A is recruited to the TIMP3 promoter and silences its expression is not clear. It is likely that a transcription factor recruits KDM1A to the TIMP3 promoter. Interestingly, additional epigenetic regulators are also involved in silencing TIMP3 gene expression in tumors. For example, EZH2 accelerates cancer cell migration by repressing TIMP3 expression in NSCLC cells [34]. TET1, a dioxygenase involved in cytosine demethylation, activates TIMP3 expression in breast cancer cells [18]. Hence, TIMP3 suppression or activation may be mediated by a multi-protein complex that includes KDM1A, EZH2, and TET1 proteins. However, how these factors may work together and be recruited to the TIMP3 promoter is not well understood and requires further study.

Overall, this study reveals a key target of KDM1A and a signaling pathway involving KDM1A-induced invasion and migration in NSCLC cells, and suggests that pharmacological inhibition of KDM1A may be used to treat NSCLC.

## MATERIALS AND METHODS

### Ethics

All animal experiments were performed using male BALB/C nude mice (4–5 weeks old). The mice were purchased from the SLAC Laboratory Animal Center (Shanghai, China) and cared for in accordance with the National Institutes of Health Guide for the Care and Use of Laboratory Animals. All animal experimental protocols performed in this study were approved by the Institutional Animal Care and Use Committee at Tongji University.

### Cell culture

Human NSCLC cell lines, PC9 and A549, were grown in DMEM medium (HyClone). NSCLC cell lines, including H1650, H292, H1975, 95D, and HCC827, were grown in RPMI-1640 medium (Hyclone). Culture media contain 10% FBS (Gibco) supplemented with penicillin (100 U/ml) and streptomycin (100 mg/ml) (Life Technologies). The cells were incubated at 37°C in a humidified atmosphere of 5% CO_2_.

### siRNA or overexpression plasmid transfection

KDM1A siRNAs were synthesized by Ribobio Inc. (Guangzhou, China). Transfections were performed with Lipofectamine 2000 (11668019, Invitrogen, CA, USA) according to the manufacturer's protocol. Total RNA or cell lysates were prepared 48 hours after transfection and were used for real-time RT-PCR or Western blotting.

The sequences for the siRNAs against KDM1A were as follows: #1: 5′-CCACGAGUCAAACCUUUA UdTdT 3′ (sense) and 5′-AUAAAGGUUUGACUCG UGGdTdT-3′ (antisense); and #2: 5′-GCUGCAGGAUCA UCUGGAAdTdT 3′ (sense) and 5′-UUCCAGAUGAUC CUGCAGCdTdT-3′ (antisense).

TIMP3 siRNAs were synthesized by GenePharma Inc. (Shanghai, China). The sequences for the siRNAs against TIMP3 were as follows: #1: 5′-GCCUUAAGCU GGAGGUCAATT-3′ (sense) and 5′-UUGACCUCCAGCU UAAGGCTT-3′ (antisense); #2: 5′-GGUAUCACCUGG GUUGUAATT-3′ (sense) and 5′-UUACAACCCAGGU GAUACCTT-3′ (antisense); and #3: 5′-CCGACAUGCUC UCCAAUUUTT-3′ (sense) and 5′-AAAUUGGAGAGC AUGUCGGTT-3′ (antisense).

The pMSCV-KDM1A plasmid was a kind gift from Yang Shi (Harvard University). KDM1A and TIMP3 overexpression plasmids and the control plasmid (GV141) were purchased from GeneChem Inc. (Shanghai, China). Plasmids were transfected into cells using Lipofectamine 2000 (11668019, Invitrogen) or DNA transfection reagent (B35101, Biotools Inc.) according to the manufacturer's protocol.

### Establishment of stable KDM1A knockdown cell lines

PC9 and A549 cells were seeded in 24-well plates. When the cell density reached 30–50% confluence, 3 ml of the lentiviral supernatant containing the lentiviral construct for sh-KDM1A or the control vector (prepared by Target Inc., Shanghai, China) was added to the cells. After 3 days of infection, cells were trypsinized and plated into new 24- well plates until the cell density reached 30–40%. The next day, culture medium containing 0.5 μg/ml puromycin was added to the cells. After 4 days of puromycin selection, cells were trypsinized and plated in 96-well plates with a target density of 1 to 3 cells in one well. Culture medium containing 0.125 μg/ml puromycin was used for continued selection. After 2 to 3 weeks, many single colonies had formed. These were initially transferred into 24-well plates and then later into 6-well plates for further expansion. Total RNA and cell lysates from these colonies were prepared and used for real time RT-PCR or Western blotting to validate the KDM1A knockdown effect in these cells.

The oligonucleotide sequences used to make the shRNA against KDM1A were as follows: forward oligo: GATCCGGCAAAGAAGCATCTGAAGTAAAG GTACCTTTACTTCAGATGCTTCTTTGTTTTTG and Reverse oligo: AATTCAAAAACAAAGAAGCATCTG AAGTAAAGGTACCTTTACTTCAGATGCTTCTTTGC CG. The two oligos were annealed and cloned into the lentiviral vector. The KDM1A-targeting sequence within these oligos is: CAAAGAAGCATCTGAAGTAAA.

### Cell proliferation assay

Cells were seeded into 96-well plates at a density of 1000 cells per well. For different treatment conditions described in the paper, each condition was replicated 6 times. At different time points, MTS cell proliferation assays were performed using CellTiter 96 AQueous One Solution Cell Proliferation Assays (MTS) (Promega G3580) according to the manufacturer's instructions. Alternatively, cell numbers were counted after proper dilutions.

### Cell invasion assay

Transwell inserts with 8 μm-pore-size membranes (BD 353097) were coated with 100 μl of diluted (1:8 in serum-free medium) Matrigel (BD Biosciences, 356234) in 24-well plates (BD353504). Cells were trypsinized and re-suspended in medium with 1% FBS. A total of 5 × 10^4^ cells in 100 μl of medium were plated in the upper chamber. The lower chamber was filled with 600 μl of medium containing 20% FBS as an attractant. Cells were incubated at 37°C under 5% CO_2_ for 48 hours, and non-invading cells were removed from the upper chamber of each transwell with a cotton swab. Invading cells attached to the lower surface of the membrane were stained with 0.1% crystal violet in methanol for 30 min at 37°C and then washed twice with PBS. Stained cells were viewed under a microscope (100 × magnification), and the number of migrated cells was counted in the whole field. The assays were performed in triplicates.

### Cell migration assay

Cells were cultured in 6-well plates until the cell density reached 80% confluence. A horizontal scratch was created using a sterile 10 μL pipette tip. The cells were washed with phosphate-buffered saline (PBS) to remove cell debris. Three different areas in each dish were selected to compare the distance that the cells had migrated from the scratch border. Images were captured at 0, 24, 48, and 72 hours to assess the extent of cell migration.

### Colony formation assay

Cells were seeded into 10-cm dishes at a density of 1000 cells per dish and cultured in the presence of puromycin (0.125 μg/ml) for 12 days. Cells were then fixed in ice-cold methanol and stained with crystal violet solutions. Bottoms of the dishes were photographed, and the numbers of visible colonies were counted by eye. The assays were performed in triplicate.

### Soft agar assay

Sterilized 4% (w/v) low-melting gel was mixed with the culture medium at a ratio of 1:3 and then added to 6-well plates. After this gel layer had solidified, the 4% low-melting gel was mixed with a cell suspension at a ratio of 1:9 and then added gently on top of the lower gel layer. After the top gel layer had solidified at room temperature, the 6-well plate was incubated at 37°C under 5% CO_2_. After two weeks, the colonies were counted.

### Cell cycle analysis

Cells were centrifuged, washed twice with PBS, and incubated with cold 70% ethanol at 4°C overnight. Cells were mixed with PI-RNase staining buffer (BD Pharmingen, #550825) according to the manufacturer's instructions. Stained cells were analyzed using a BD Accuri C6 Flow Cytometer. Results were plotted using FlowJo 7.6.1 software.

### Plasmid cloning and luciferase assay

A 1.2 kb length of the upstream region of the TIMP3 promoter was cloned into the pGL3-basic luciferase construct. Luciferase reporter assays were performed using a dual-luciferase reporter assay system (E1910, Promega), according to the manufacturer's instructions.

### Western blotting (WB)

Cells were lysed in RIPA lysis buffer (#P0013B, Beyotime Biotechnology). Total protein in the cell lysates was quantified using a BCA protein quantification kit (WB0124, Shanghai WEIAO Bio Tech). An equal amount of protein (40–50 μg) from each condition was separated by 10% SDS-PAGE, electrotransferred onto a 0.45 μM NC membrane (#HATF00010, Millipore), and incubated overnight at 4°C with a primary antibody (1:1000 dilution) and then incubated with a secondary antibody (1:3000 dilution). GAPDH was used as a loading control. Protein bands were visualized using ECL chemiluminescent reagent (#34080 and #34095, Thermo) and detected by a ChemiDOC MP Imaging System (BIO-RAD). The following primary antibodies were used: KDM1A (CST, #2184), TIMP3 (Santa Cruz, #6836), MMP2 (Santa Cruz, #13594), H3K4me2 (CST, #9725), total histone H3 (CST, #4499), SAPK/JNK(CST, #9252), Phospho-SAPK/JNK (Thr183/Tyr185) (CST, #9251), MAO-A/B (Santa Cruz, #50333), caspase 3 (CST, #9665), and PARP (CST, #9542), GAPDH (Sigma-Aldrich, #G9545), p-EGFR (Tyr1068) (CST #3777), EGFR (CST #4267), β-Actin (CST, #4970). The following secondary antibodies were used: anti-rabbit IgG (CST, #7074S) and goat polyclonal anti-mouse IgG (Abcam, #ab136815).

### Apoptosis assay

Cells were washed twice in cold 1 × PBS twice. Then, 1 × 10^6^ cells per ml were resuspended in 1 × binding buffer, and 100 μl of the cell suspension was mixed with 5 μl FITC and 5 μl PI using the FITC Annexin V apoptosis detection kit (BD 556547), according to the manufacturer's instructions. Stained cells were analyzed using an Accuri C6 flow cytometer (BD Biosciences, USA).

### RNA isolation and real-time RT-PCR

Total RNA from tissues and cells was extracted using an RNAprep Pure Cell kit (#DP430, TIANGEN) and quantified using a Nanodrop. A total of 3 μg of total RNA was reverse-transcribed into cDNA using a RevertAid First Strand cDNA Synthesis Kit (#K1622, Thermo, USA). cDNA was diluted to a ratio of 1:100 or 1:1000 using EB buffer. Real-time PCR was performed by mixing the diluted template and primers with a real-time SYBR Green PCR kit (#L01–160, BiovisuaLab, China) and detected by a real-time PCR detection system (ABI 7500). The relative gene expression level was normalized to GAPDH. The PCR primers used were as follows: KDM1A forward, 5′-ATCTGCAGTCCAAAGGATGG-3′; KDM1A reverse, 5′-GCCAACAATCACATCGTCAC-3′; GAPDH forward, 5′-CCGGGAAACTGTGGCGTGATGG-3′; GAPDH reverse, 5′-AGGTGGAGGAGTGGGTGTCGCTGTT-3′; TIMP3 forward, 5′-CTGACAGGTCGCGTCTATGA-3′; and TIMP3 reverse, 5′-GCAAGGCAGGTAGTAGC AGG-3′.

### Chromatin immunoprecipitation (ChIP)-qPCR

ChIP experiments were performed as previously described [35]. The updated protocol can be found at http://research.hudsonalpha.org/Myers/. Anti-H3K4me2 (ab7766) and anti-KDM1A (ab17721) antibodies were used, and IgG was used as the control antibody. Purified DNA was resuspended in EB buffer for subsequent SYBR green-based real-time PCR. The following primers, which cover the TIMP3 promoter and upstream regions, were used: TIMP3(−685)-Forward, TCTGCTGTCCCAATGTCACT; TIMP3(−685)-Reverse, CTTTCCCTTTCTCTTCCGCT; TIMP3(−506)-Forward, GGCCATTTGCAGTTAGATGG; TIMP3(−506)-Reverse, AAAGATGGGATGATTGGTGG; TIMP3(−345)-Forward, TGTGCTTGCTTTTCCTACCC; and TIMP3(−345)-Reverse, TTGACATCTTGATTCTGCTTTG; TIMP3 (−180)-Forward, TGGATCGTTCTCCTGGTTTT; TIMP3 (−180)-Reverse, CAAGAGGAAGTGGTTCCCTG; TIMP3 (−43)-Forward, AGGGTCTTTGCACTTGCTGT; TIMP3 (−43)-Reverse, GCGATAGTAAGGGAAGGGCT; TIMP3 (+65)-Forward, AGCCCTTCCCTTACTATCGC; TIMP3 (+65)-Reverse, TCAACAAACACGGTTCAGGA;

### *In vivo* tumorigenesis and metastasis experiments

For the xenograft tumor growth assay, a total of 2 × 10^6^ cells suspended in 100 μl PBS were injected subcutaneously into the right axillary region of 5-week-old mice. Tumor size was measured every 5 to 7 days using a digital caliper, and tumor volume (v) was calculated based on this formula: v = 0.5*a*b^2^, where a = long diameter and b = short diameter. After 5 weeks, xenograft tumor tissues were isolated, photographed, and stored in liquid nitrogen for gene expression microarray analysis.

For mice foot pad injection experiments, 5-week-old male nude mice were divided into two groups, with 10 mice in each group: the treatment group was injected with 200 μl 1× PBS containing 2 × 10^6^ PC9 cells stably expressing KDM1A shRNA into the hind foot pads; the control group was injected with the same amount of PC9 cells expressing the control shRNA. After four weeks, mice were sacrificed by cervical vertebral dislocation and dissected, and visible metastatic nodules, such as lymph nodes, were isolated and fixed in 4% paraformaldehyde, which was then paraffin-embedded, sliced, and subjected to hematoxylin and eosin staining (H&E staining). The H&E-stained slides were scanned using a Leica SCN400 and analyzed using SlidePath Gateway LAN software.

For left ventricle injection experiments, the procedure was essentially the same as used for the footpad injection experiment, except as follows: 3 × 10^5^ cells in 100 μl 1xPBS were injected into the left ventricles of the mice.

For *in vivo* 2-PCPA experiment, PC9 cells derived xenograft tumors were allowed to grow to around 100 mm^3^, then mice were divided into two groups, one group was treated with 10 mg/kg 2-PCPA, the control group was treated with the same volume of 1xPBS, by intragastric injection. Tumor volumes were measured every three days until mice were sacrificed at week seven, then tumor tissues were isolated, photographed, and weighted.

### Small molecule inhibitors

Gefitinib was purchased from Selleck (#S1025). Tranylcypromine (also Parnate or 2-PCPA) was purchased from Enzo Life Science (#EI-217). PC9 and A549 cells were plated in 6-well plates until the cell density reached 30–40% confluence, and the cells were then treated with different concentrations of 2-PCPA for 48 hours. Cell lysates were prepared and used for WB analysis. In addition, treated cells were also used in the cell proliferation, invasion and migration assays.

### Statistical analysis

The RNA-seq data of lung adenocarcinoma (LUAD) were obtained from the TCGA project (http://cancergenome.nih.gov) and the firebrowse (http://firebrowse.org). All values were transformed to a log 2 scale. Quantile normalization was used to process and normalize the RNA-seq data. Pearson's correlation was applied to analyze the correlation coefficient between two genes in the normal or cancer samples. Differentially expressed genes were analyzed by DESeq in R (http://bioconductor.org/packages/release/bioc/html/DESeq.html) based on the raw RSEM counts of the genes in cancer and normal samples. Gene expression and survival information for NSCLC patients were from the released database (2015 version) downloaded from http://kmplot.com/ [28]. Survival analysis was performed using the logrank test and Kaplan-Meier plots in the statistics package for IBM SPSS version 22. For comparisons between two groups, student's *t* test was used. For comparisons among multiple groups, one-way anova was used. For all analyses, a *p* value of < 0.05 was considered statistically significant. *P < 0.05, **P < 0.01, ***P < 0.001, ****P < 0.0001.

## SUPPLEMENTARY MATERIALS FIGURES AND TABLES




