# Supplementary material for: KDM1A promotes tumor cell invasion by silencing TIMP3 in non-small cell lung cancer cells
Source: Oncotarget. 2016 Apr 2;7(19):27959–74. doi: 10.18632/oncotarget.8563 (PMC5053702; doi:10.18632/oncotarget.8563)
Supplement: Supplementary file 1 [file oncotarget-07-27959-s001.pdf]

KDM1A promotes tumor cell invasion by silencing TIMP3 in non-small cell lung cancer cells

Supplementary Materials

MATERIALS AND METHODS

Microarray and computational analysis

Total RNA, including small RNA from xenograft tissues, was extracted using a miRNeasy Mini kit (Qiagen, #217004). RNA was amplified into cDNA using an Ambion WT expression kit (Life technologies, #4411973). Sense-strand cDNA was fragmented and labeled using an Affymetrix GeneChip WT Terminal Labeling kit (Affymetrix, #900671). Subsequently, labeled DNA was hybridized to an Affymetrix GeneChip Human Transcriptome Array 2.0 using a GeneChip Hybridization, Wash, and Stain Kit (Affymetrix, #900720). The GeneChip Human Transcriptome Array 2.0 covers over 245,000 coding transcripts and over 40,000 non-coding transcripts. After washes, the array was scanned by a GeneChip Scanner 3000 7G (Affymetrix), and array data were analyzed using Affymetrix Expression Console Software and Transcriptome Analysis Console (TAC) Software.

Microarray data were normalized using the updated RMA sst (signal space transformation) algorithm in an Affymetrix Expression Console. One-way between-subject ANOVA (unpaired) tests were used to identify genes that were differentially expressed between the control and experiment groups. After the significance and FDR analyses, we selected the differentially expressed genes according to p-value thresholds. A p value < 0.05 was considered a significant difference [2–4]. Hierarchical clustering (Cluster3.0) and TreeView analysis (Stanford University, USA) were performed based on the results for the differentially expressed genes.

The microarray data discussed in this paper have been deposited in NCBI's Gene Expression Omnibus (GEO) and are accessible through GEO Series accession number GSE71358 (http://www.ncbi.nlm.nih.gov/geo/query/acc.cgi?acc = GSE71358).

GO analysis

The Database for Annotation, Visualization and Integrated Discovery (DAVID) v6.7 was used to identify the significant biological processes that were associated with the differentially expressed genes in both the control and experimental groups [5, 6].

REFERENCES

1. Gyorffy B, Surowiak P, Budczies J, Lanczky A. Online survival analysis software to assess the prognostic value of biomarkers using transcriptomic data in non-small-cell lung cancer. PLoS One. 2013; 8:e82241.
2. Wright GW, Simon RM. A random variance model for detection of differential gene expression in small microarray experiments. Bioinformatics. 2003; 19:2448–2455.
3. Yang H, Crawford N, Lukes L, Finney R, Lancaster M, Hunter KW. Metastasis predictive signature profiles pre-exist in normal tissues. Clin Exp Metastasis. 2005; 22:593–603.
4. Clarke R, Ressom HW, Wang A, Xuan J, Liu MC, Gehan EA, Wang Y. The properties of high-dimensional data spaces: implications for exploring gene and protein expression data. Nat Rev Cancer. 2008; 8:37–49.
5. Huang da W, Sherman BT, Lempicki RA. Systematic and integrative analysis of large gene lists using DAVID bioinformatics resources. Nat Protoc. 2009; 4:44–57.
6. Huang da W, Sherman BT, Lempicki RA. Bioinformatics enrichment tools: paths toward the comprehensive functional analysis of large gene lists. Nucleic Acids Res. 2009; 37:1–13.

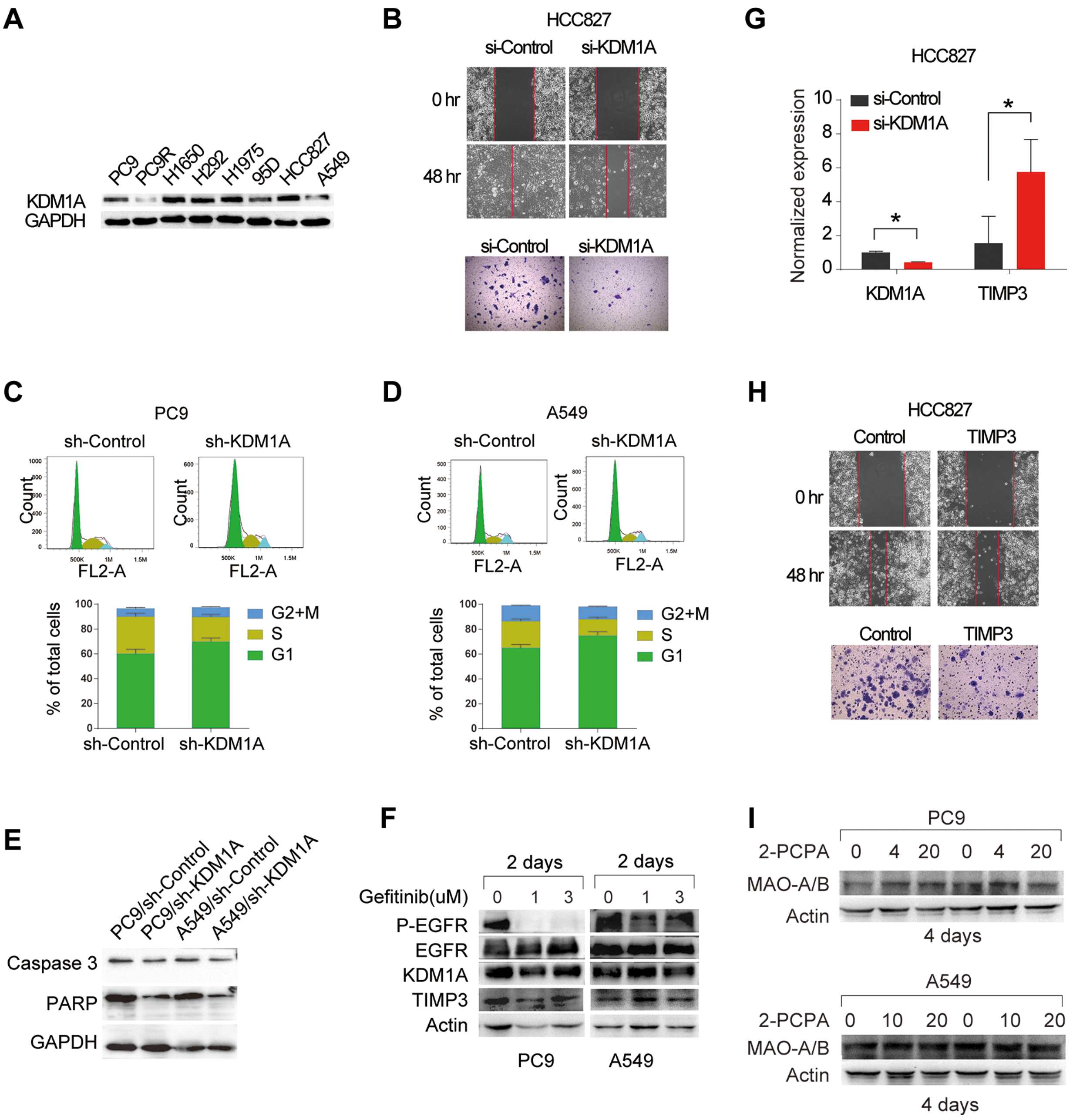

Supplementary Figure S1: Additional characterization of KDM1A expression, function, and regulation in NSCLC cell lines. (**A**) WB detection of KDM1A expression in the following lung cancer cell lines: PC9, PC9R, H1650, H292, H1975, 95D, HCC827, and A549. PC9R is PC9 derived cells that are resistant to gefitinib. GAPDH serves as a loading control. (**B**) Cell migration (top panel) and invasion (bottom panel) assays of HCC827 cells transiently transfected with the control siRNA (si-Control) or KDM1A siRNA (si-KDM1A). The red line indicates the edge of migrating cells at a given time point. (**C**, **D**) PC9 (C) or A549 (D) cells stably expressing the control shRNA (sh-Control) or KDM1A shRNA (sh-KDM1A) were subjected for cell cycle analysis. The histograms show the representative results generated by FlowJo 7.6.1 software (upper panel). The bar graphs show percentages of cells within each cycle phase (G1, S, G2, and M) for all conditions (lower panel). Each condition was performed in triplicates. Data are represented as mean (SD). (**E**) PC9 or A549 cells stably expressing the control shRNA (sh-Control) or KDM1A shRNA (sh-KDM1A) were subjected to WB analysis using antibodies against apoptosis markers, including caspase 3 and PARP. GAPDH serves as the loading control. (**F**) WB detection of phosphorylated (P-EGFR) or total EGFR, KDM1A, and TIMP3 expressions in PC9 (left panel) or A549 cells (right panel) treated with 0, 1, or 3 µM of gefitinib for 2 days. Actin serves as a loading control. (**G**) Normalized KDM1A and TIMP3 mRNA expressions in HCC827 cells transiently transfected with the control siRNA (si-Control) or KDM1A siRNA (si-KDM1A), detected by real time RT-PCR. Each condition was performed in triplicates. Data are represented as mean (SD). (**H**) Cell migration (top panel) and invasion (bottom panel) assays of HCC827 cells transiently transfected with the control (Control) or TIMP3 (TIMP3) overexpression plasmid. (**I**) WB detection of MAO-A/B expression in PC9 (left panel) and A549 (right panel) cells treated with 0, 4, 10 or 20 µM of 2-PCPA for 4 days. Actin serves as a loading control. WB detection was performed in replicates.

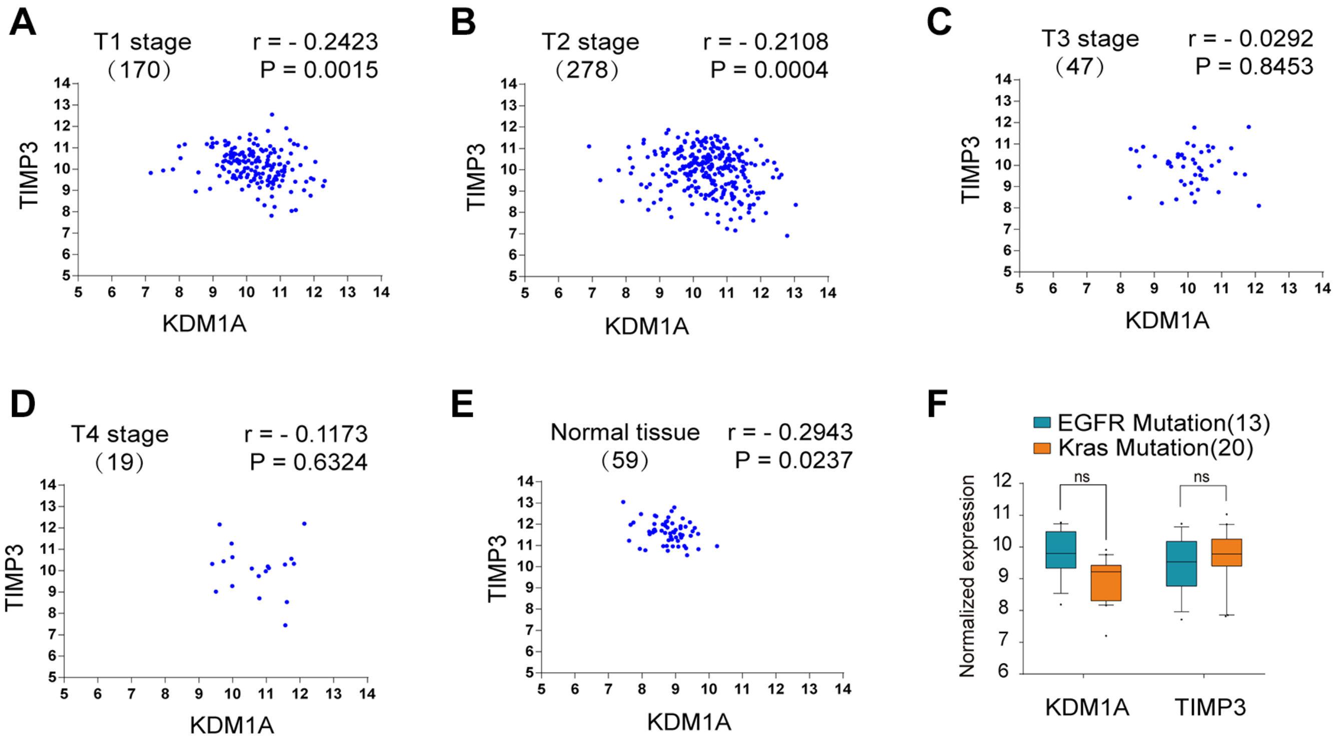

Supplementary Figure S2: Correlation analysis between KDM1A and TIMP3 expressions in different stages of NSCLC. (**A**) T1 stage: $r = –0.2423$, $P = 0.0015$. (**B**) T2 stage: $r = –0.2108$, $P = 0.0004$. (**C**) T3 stage: $r = –0.02925$, $P = 0.8453$. (**D**) T4 stage: $r = –0.1173$, $P = 0.6324$. (**E**) Normal tissues: $r = –0.2943$, $P = 0.0237$. Numbers within the parentheses represent the sample size. (**F**) Comparison of normalized KDM1A and TIMP3 expressions in NSCLC carrying EGFR mutations (EGFR mutation, in the green color) and Kras mutations (Kras mutation, in the orange color). All the original data are from LUAD of the TCGA database. The number within the parenthesis represents the sample size.

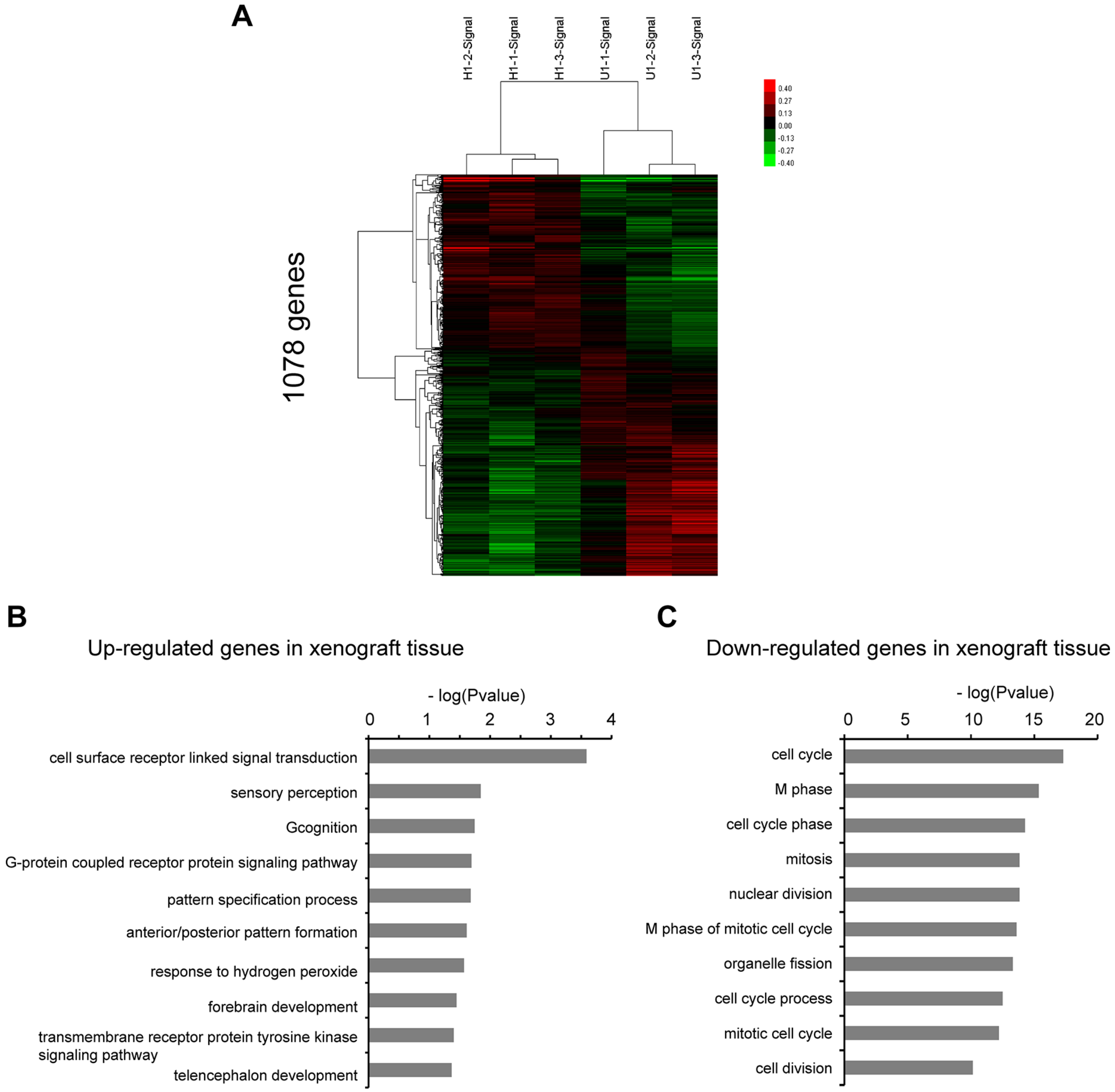

Supplementary Figure S3: Clustered heatmap and pathway analysis of differentially expressed mRNAs in xenograft tissues. (**A**) The hierarchical clustering was based on 1078 differentially expressed genes between two groups including the KDM1A shRNA group (H1–1, H1–2, H1–3) and the control shRNA group (U1–1, U1–2, U1–3) (P < 0.05). Fold-changes in gene expression between two groups are expressed by a color gradient intensity scale, as shown in the right-top corner. The green color indicates down-regulation, and the red color indicates up-regulation of gene expression in the KDM1A shRNA group. Each column represents a separate sample, and each row represents a single gene. (**B**, **C**) Biological processes enriched in up-regulated (B) or down-regulated (C) genes in xenograft tumors derived from PC9 cells stably expressing KDM1A shRNA compared to those expressing the control shRNA. The x-axis represents – log (P value), and the y-axis represents names of the pathways. GO analysis was performed using the DAVID software.

Supplementary Table S1: The microarray analysis of differentially expressed genes in xenograft tissues. (**A**) List of 2994 differentially expressed genes, including lncRNAs, in xenograft tissues derived from PC9 cells stably expressing KDM1A shRNA vs. those expressing the control shRNA ($P < 0.05$). (**B**) List of 1078 differentially expressed protein-coding genes, with normalized array intensities from six xenograft tissues. (**C**, **D**) GO Term BP (biological process) analysis of up-regulated genes (C) or down-regulated genes (D) in xenograft tissues derived from PC9 cells stably expressing KDM1A shRNA, compared to those expressing the control shRNA.

Supplementary Table S2: NSCLC patient survival analysis based on KDM1A and TIMP3 expressions

A

Sample	KDM1A Expression (1 = high)	Time (months)	Event (1 = Death)
1	0	107.7333333	0
2	1	5.433333333	1
3	0	18.76666667	1
4	0	10.26666667	1
5	1	19.9	1
6	1	2.066666667	1
7	0	20.16666667	1
8	0	61.3	1
9	0	17.43333333	1
10	0	13.33333333	1
11	1	8.5	1
12	1	16.56666667	1
13	1	2.766666667	1
14	0	12	1
15	0	2.933333333	1
16	1	9.7	1
17	0	5.066666667	1
18	1	71.26666667	1
19	0	117.3333333	1
20	0	136.3333333	1
21	0	110.2666667	1
22	1	17.43333333	1
23	0	193.1666667	0
24	0	9.5	1
25	0	11.63333333	1
26	0	108.9666667	1

27	1	20	1
28	0	125.7666667	1
29	1	130.0333333	0
30	1	112.6666667	1
31	1	125.9333333	0
32	1	122.4333333	0
33	1	113.9666667	0
34	1	96.2	1
35	1	0.2	1
36	1	6.633333333	1
37	1	11.86666667	1
38	1	2.666666667	1
39	0	133.5666667	1
40	0	159.0333333	0
41	1	8.333333333	1
42	1	92.96666667	1
43	0	10.9	1
44	0	151.8333333	0
45	0	151.7333333	0
46	1	13.43333333	1
47	1	12.56666667	1
48	0	8.9	1
49	0	18.03333333	1
50	1	63.4	1
51	0	6.033333333	1
52	0	171.8666667	0
53	1	81.1	1
54	0	69.93333333	1
55	0	119.8666667	1
56	0	41.36666667	1
57	0	109.1333333	0
58	1	34.86666667	1
59	1	42.06666667	1
60	0	37.96666667	1
61	0	96.93333333	0
62	1	29	1
63	0	93.43333333	0
64	0	28.33333333	1
65	0	40.3	1
66	0	72.33333333	1

67	1	87.56666667	0
68	0	79.5	0
69	1	78.06666667	0
70	0	40.76666667	1
71	1	78.33333333	0
72	0	82.76666667	0
73	1	33.83333333	1
74	1	41.36666667	1
75	1	49.46666667	1
76	1	1.733333333	1
77	0	10.76666667	1
78	0	65.23333333	0
79	1	61.73333333	0
80	1	25.53333333	1
81	1	62.86666667	0
82	1	34.2	1
83	1	50.73333333	1
84	1	39.93333333	1
85	0	160.9666667	0
86	0	34.03333333	1
87	0	54.46666667	1
88	1	46.2	1
89	1	137.6	0
90	1	25.93333333	1
91	1	130.1	0
92	1	41.23333333	1
93	1	33.1	1
94	1	108.8	0
95	1	57	1
96	1	73.3	1
97	1	171.2	0
98	0	165.6333333	0
99	1	48.9	1
100	1	68.66666667	1
101	1	50.33333333	1
102	0	176.1	0
103	1	95.06666667	1
104	1	37.6	1
105	1	85.06666667	0

106	0	34.33333333	1
107	0	221	0
108	0	90	0
109	1	88	0
110	0	82	0
111	1	23	1
112	1	52	1
113	1	75	0
114	1	49	1
115	0	76	0
116	1	72	0
117	0	64	0
118	0	67	0
119	0	60	0
120	1	81	0
121	1	34	1
122	1	78	0
123	1	69	0
124	0	59	0
125	1	61	0
126	1	77	0
127	0	69	1
128	1	13	1
129	0	80	0
130	1	70	0
131	1	62	0
132	0	76	0
133	0	68	1
134	0	23	1
135	0	48	1
136	1	35	1
137	1	72	1
138	1	66	0
139	1	65	0
140	0	4	0
141	1	88	0
142	1	52	1
143	1	59	1
144	1	76	0

145	1	15	1
146	1	31	1
147	1	75	0
148	1	79	0
149	0	69	1
150	1	65	0
151	1	39	1
152	1	42	1
153	1	62	0
154	0	73	0
155	1	11	1
156	1	35	0
157	1	46	1
158	0	1	0
159	1	70	0
160	1	76	1
161	1	63	1
162	0	60	0
163	0	62	0
164	1	50	0
165	0	38	1
166	1	12	1
167	1	59	0
168	1	12	1
169	1	37	1
170	1	24	1
171	0	63	0
172	1	69	0
173	1	66	0
174	1	66	0
175	1	55	0
176	1	59	0
177	1	10	1
178	0	23	1
179	1	93	0
180	1	52	0
181	1	63	0
182	0	25	1
183	0	57	1

184	0	36	1
185	1	38	1
186	1	65	0
187	1	37	0
188	0	8	1
189	0	22	1
190	1	39	0
191	0	7	1
192	1	62	0
193	1	47	1
194	0	41	0
195	0	18	1
196	1	35	1
197	1	32	0
198	1	60	0
199	1	80	0
200	1	70	0
201	1	131	0
202	0	6	0
203	0	48	1
204	1	18	0
205	1	94	0
206	1	72	0
207	1	21	0
208	1	9	0
209	1	60	0
210	0	15	0
211	0	2	0
212	0	16	0
213	0	10	1
214	1	61	0
215	1	1	0
216	1	41	0
217	1	18	1
218	1	23	1
219	0	19	1
220	1	89	0
221	0	7	1
222	1	50	0

223	1	62	0
224	1	7	1
225	0	33	0
226	1	15	0
227	1	91	1
228	1	15	1
229	1	54	0
230	0	14	1
231	1	4	1
232	0	99	0
233	1	1	1
234	0	50	0
235	1	8.52	0
236	0	8.23	0
237	1	0.03	1
238	1	8.6	0
239	1	1.59	1
240	1	6.09	1
241	1	0.19	1
242	1	6.93	0
243	1	7.71	0
244	1	0.9	1
245	1	3.74	1
246	0	7.2	0
247	0	5.87	0
248	1	5.42	0
249	1	5.03	0
250	1	2.28	1
251	1	1.86	1
252	1	2.82	1
253	0	4.46	0
254	0	3.95	0
255	0	3.51	0
256	1	4.89	0
257	1	4.76	1
258	1	6.74	0
259	1	5.38	0
260	1	2.04	1
261	1	6.03	1

262	0	127	0
263	0	12.57	1
264	0	0.67	1
265	0	98.93	0
266	0	87.7	1
267	0	10.33	1
268	0	4.87	1
269	1	20.37	1
270	0	66.47	1
271	1	110.8	0
272	0	22.57	1
273	0	103.7	0
274	1	124.83	0
275	0	21.3	1
276	1	0.87	1
277	0	8.27	1
278	0	116.83	0
279	0	17.3	1
280	0	79.87	1
281	0	16.7	1
282	0	24.17	1
283	1	46.7	1
284	0	2.53	1
285	0	63.87	0
286	0	27.97	0
287	1	89.73	0
288	0	110.8	0
289	0	20.53	1
290	1	67.87	0
291	1	20.93	0
292	1	23.13	0
293	1	20.37	1
294	1	5.53	1
295	0	13.73	1
296	0	124.3	0
297	0	89.03	0
298	0	89.03	0
299	0	78.63	0
300	1	60.73	1

301	1	2.5	1
302	0	7.13	1
303	0	59.83333333	0
304	0	11.6	1
305	1	34.8	0
306	0	35.66666667	0
307	1	43.36666667	0
308	1	60.76666667	0
309	1	33.8	1
310	1	58.53333333	0
311	1	33.56666667	1
312	0	24.1	0
313	1	39.6	1
314	0	35.2	0
315	1	42.8	1
316	1	68.33333333	0
317	0	16.93333333	1
318	1	20.3	1
319	1	73.33333333	0
320	1	63.53333333	0
321	1	21.33333333	1
322	0	54.3	0
323	1	22.56666667	1
324	1	19.16666667	1
325	1	49.5	0
326	1	49.43333333	1
327	1	4.9	1
328	1	12.46666667	1
329	1	70.86666667	0
330	1	1.066666667	1
331	1	55.36666667	1
332	0	42.63333333	0
333	0	59.46666667	0
334	1	11.16666667	1
335	1	27.2	1
336	1	28.36666667	1
337	0	51.23333333	0
338	1	12.33333333	0
339	1	37.5	0

340	0	29.13333333	1
341	0	1.9	1
342	1	24.33333333	1
343	1	41.66666667	0
344	1	41.63333333	0
345	1	24.13333333	1
346	1	19.83333333	1
347	1	39.83333333	0
348	0	6.5	1
349	1	39	1
350	1	7	1
351	1	48	0
352	1	31	0
353	1	6	0
354	1	3	1
355	1	2	1
356	0	24.5	1
357	1	11	1
358	1	9	1
359	1	27	0
360	1	10.5	1
361	0	27.17043121	0
362	0	33.83983573	0
363	0	34.7597536	0
364	0	25.56057494	0
365	0	32.65708418	0
366	0	12.12320328	1
367	0	31.08008214	0
368	0	42.38193018	1
369	0	39.09650924	0
370	0	35.38398358	0
371	0	40.21355236	1
372	0	26.34907597	0
373	0	49.7412731	0
374	0	40.7063655	0
375	0	40.60780288	0
376	0	32.95277207	1
377	0	50.1026694	0
378	0	24.24640657	0

379	0	32.42710472	0
380	0	5.585215608	0
381	0	7.786447644	0
382	0	11.36755646	0
383	0	35.67967146	0
384	0	46.42299794	0
385	0	26.0862423	1
386	0	25.95482546	1
387	0	58.02053388	0
388	0	28.8788501	1
389	0	43.07186858	1
390	0	69.71663244	0
391	1	121	0
392	1	21	1
393	1	75	0
394	1	165	0
395	1	68	0
396	0	85	0
397	1	87	0
398	0	51	0
399	0	34	1
400	1	20	1
401	0	43	0
402	0	29	1
403	1	58	0
404	0	102	0
405	0	8	1
406	0	89	1
407	0	131	0
408	0	101	0
409	0	25	1
410	1	67	0
411	1	83	0
412	1	96	0
413	0	1	0
414	1	36	0
415	0	175	1
416	1	182	0
417	0	32	0

418	0	74	1
419	0	62	0
420	0	25	1
421	1	90	1
422	1	40	1
423	0	85	0
424	0	113	0
425	0	12	1
426	1	72	0
427	0	42	0
428	1	60	0
429	1	52	1
430	0	72	0
431	1	14	1
432	1	13	1
433	0	0	1
434	1	153	0
435	0	18	0
436	0	38	1
437	1	150	1
438	1	28	1
439	1	76	0
440	1	127	1
441	0	18	1
442	1	108	0
443	0	43	0
444	1	142	0
445	1	127	0
446	1	175	1
447	0	57	0
448	0	61	0
449	1	65	0
450	0	104	0
451	0	115	0
452	1	2	1
453	0	13	1
454	0	14	1
455	1	134	0
456	1	0	1

457	1	11	1
458	0	74	0
459	1	29	0
460	0	163	0
461	0	107	1
462	1	99	1
463	0	21	1
464	1	4	1
465	1	23	1
466	1	17	1
467	0	103	1
468	0	42	1
469	1	55	1
470	1	179	1
471	1	23	1
472	1	174	0
473	1	26	1
474	1	3	0
475	0	14.56666667	1
476	1	24.76666667	0
477	0	86.7	0
478	0	39.1	0
479	0	78.96666667	0
480	0	62.63333333	0
481	0	20.2	0
482	1	10	1
483	0	63.96666667	0
484	0	61.16666667	0
485	0	60.93333333	0
486	0	61.36666667	1
487	0	21.3	1
488	0	43.7	1
489	0	36.6	0
490	0	39.63333333	0
491	1	128.7666667	0
492	0	49.16666667	0
493	0	61.86666667	0
494	0	102.2333333	0
495	1	38.06666667	0

496	0	60.83333333	0
497	0	53.66666667	0
498	1	67.13333333	0
499	0	60.86666667	0
500	0	73.03333333	0
501	1	111.4	0
502	0	44.26666667	0
503	1	97.5	0
504	0	96	0
505	1	57.46666667	0
506	0	92.16666667	0
507	1	50.96666667	0
508	1	7.366666667	1
509	0	18.2	1
510	0	11.53333333	1
511	0	28.43333333	1
512	0	88.7	1
513	0	27.23333333	0
514	1	14.16666667	1
515	0	42.7	0
516	1	34.8	0
517	0	18.83333333	0
518	0	35.3	1
519	0	54.16666667	1
520	0	56.5	1
521	1	99.2	0
522	0	38.1	1
523	0	56.06666667	0
524	1	41.4	1
525	0	75.06666667	0
526	0	31.43333333	1
527	0	44.86666667	1
528	0	49.4	1
529	0	79.53333333	0
530	1	52	0
531	0	33.36666667	1
532	0	8.633333333	1
533	0	76.3	0
534	0	63.86666667	0

535	0	68.26666667	0
536	0	49.16666667	0
537	0	51.3	0
538	0	21.2	0
539	1	42.5	0
540	0	66.83333333	0
541	0	65.5	0
542	0	80.83333333	0
543	0	52.2	0
544	0	33	0
545	0	24.3	0
546	0	78.46666667	0
547	0	27.26666667	0
548	0	66.96666667	0
549	1	84.96666667	0
550	0	113.7	0
551	1	60.2	0
552	0	64.56666667	0
553	0	50.66666667	0
554	0	45.83333333	0
555	0	58.53333333	0
556	1	53.23333333	0
557	0	57.13333333	0
558	0	54.56666667	0
559	0	55.1	0
560	0	39.86666667	0
561	0	56.73333333	0
562	1	68.33333333	0
563	0	41.86666667	0
564	0	27.26666667	0
565	1	38.46666667	0
566	0	101.9333333	0
567	1	64.7	0
568	1	53.2	0
569	1	81.66666667	0
570	1	62.7	0
571	1	54.56666667	0
572	0	67.8	0
573	0	71.13333333	0

574	0	25.96666667	1
575	0	18	1
576	0	43.9	0
577	0	66	0
578	0	23.4	1
579	0	18.8	1
580	0	26.26666667	1
581	0	48.76666667	0
582	0	44.83333333	1
583	0	27.73333333	0
584	1	89.56666667	0
585	1	37.4	1
586	1	88.93333333	0
587	1	64.53333333	0
588	0	29.03333333	0
589	1	89.03333333	0
590	0	49.4	0
591	0	37.6	0
592	0	34.73333333	1
593	1	40.96666667	1
594	0	75.43333333	1
595	1	59	0
596	1	47.76666667	1
597	0	60.76666667	0
598	1	66.26666667	0
599	0	47.8	0
600	0	21	1
601	0	51.23333333	0
602	0	47.63333333	1
603	1	34.6	1
604	0	82.86666667	0
605	0	24.66666667	0
606	0	34.73333333	0
607	0	42.23333333	0
608	1	33.9	0
609	1	34.7	0
610	1	61.96666667	0
611	0	57.23333333	0
612	0	49.8	0

613	1	25.13333333	0
614	0	78.8	0
615	1	57.76666667	0
616	0	64.7	0
617	1	25.8	0
618	1	97.1	0
619	0	60.1	0
620	0	63.73333333	0
621	0	49.36666667	0
622	1	41.03333333	0
623	1	65.46666667	0
624	0	111.7333333	0
625	0	71.93333333	0
626	0	115.5333333	0
627	0	108.7666667	0
628	0	60.76666667	0
629	0	61.86666667	0
630	0	81.63333333	0
631	1	61.96666667	0
632	0	66.6	0
633	0	61.73333333	0
634	0	63.13333333	0
635	0	62.03333333	0
636	0	55.63333333	0
637	0	106.3	0
638	0	60.73333333	0
639	0	60.3	0
640	1	85.06666667	0
641	1	98.93333333	0
642	0	102.2	0
643	0	47.06666667	0
644	0	43.9	0
645	1	39.33333333	0
646	1	56.26666667	0
647	0	47.23333333	0
648	1	47.53333333	0
649	0	55.8	0
650	1	61.6	0
651	1	61.86666667	0

652	0	59.33333333	0
653	0	69.46666667	0
654	1	62.63333333	0
655	1	37.13333333	0
656	0	93.53333333	0
657	1	55.43333333	0
658	0	57.53333333	0
659	1	54.3	0
660	1	72.63333333	0
661	1	63.9	0
662	0	48.2	0
663	0	38.73333333	0
664	1	41.03333333	0
665	1	31.36666667	0
666	0	54.1	0
667	1	61.83333333	0
668	1	46.8	0
669	0	74.03333333	0
670	0	28.3	0
671	1	27.7	0
672	0	107.3	0
673	1	86.16666667	0
674	0	81.4	0
675	0	63.66666667	0
676	1	61.13333333	0
677	0	97.26666667	0
678	1	76.16666667	0
679	1	71.63333333	0
680	0	85.36666667	0
681	0	64.9	0
682	0	61.36666667	0
683	0	65.7	0
684	1	80.3	0
685	0	54.13333333	0
686	0	81	0
687	1	61.86666667	0
688	0	50.53333333	0
689	1	60.63333333	0
690	1	80.43333333	0

691	1	61.63333333	0
692	1	72.7	0
693	1	72.26666667	0
694	1	71.8	0
695	0	69.1	0
696	0	66.46666667	0
697	1	71.23333333	0
698	0	66.6	0
699	0	63.6	0
700	0	55.33333333	0
701	0	48	0
702	0	39	0
703	1	21	1
704	1	15	1
705	1	33	0
706	1	44	0
707	1	20	1
708	1	47	0
709	1	39	0
710	1	61	0
711	0	20	1
712	1	42	0
713	1	43	0
714	1	52	1
715	1	45	0
716	1	34	1
717	0	3	1
718	1	44	0
719	1	50	0
720	1	10	1

B

Sample	TIMP3 Expression (1 = high)	Time (months)	Event
1	1	107.7333333	0
2	0	5.433333333	1
3	0	18.76666667	1
4	1	10.26666667	1
5	0	19.9	1
6	1	2.066666667	1
7	0	20.16666667	1
8	0	61.3	1
9	0	17.43333333	1
10	0	13.33333333	1
11	0	8.5	1
12	1	16.56666667	1
13	0	2.766666667	1
14	1	12	1
15	0	2.933333333	1
16	0	9.7	1
17	0	5.066666667	1
18	0	71.26666667	1
19	0	117.3333333	1
20	0	136.3333333	1
21	0	110.2666667	1
22	1	17.43333333	1
23	1	193.1666667	0
24	1	9.5	1
25	0	11.63333333	1
26	0	108.9666667	1
27	0	20	1
28	1	125.7666667	1
29	0	130.0333333	0
30	1	112.6666667	1
31	0	125.9333333	0
32	0	122.4333333	0
33	0	113.9666667	0
34	1	96.2	1
35	0	0.2	1
36	0	6.633333333	1
37	0	11.86666667	1
38	1	2.666666667	1

39	0	133.5666667	1
40	1	159.0333333	0
41	0	8.333333333	1
42	0	92.96666667	1
43	0	10.9	1
44	0	151.8333333	0
45	1	151.7333333	0
46	0	13.43333333	1
47	0	12.56666667	1
48	0	8.9	1
49	0	18.03333333	1
50	0	63.4	1
51	0	6.033333333	1
52	1	171.8666667	0
53	0	81.1	1
54	0	69.93333333	1
55	1	119.8666667	1
56	1	41.36666667	1
57	1	109.1333333	0
58	1	34.86666667	1
59	0	42.06666667	1
60	0	37.96666667	1
61	1	96.93333333	0
62	0	29	1
63	1	93.43333333	0
64	1	28.33333333	1
65	1	40.3	1
66	0	72.33333333	1
67	0	87.56666667	0
68	1	79.5	0
69	0	78.06666667	0
70	0	40.76666667	1
71	1	78.33333333	0
72	0	82.76666667	0
73	0	33.83333333	1
74	0	41.36666667	1
75	0	49.46666667	1
76	0	1.733333333	1
77	1	10.76666667	1

78	0	65.23333333	0
79	1	61.73333333	0
80	1	25.53333333	1
81	1	62.86666667	0
82	1	34.2	1
83	0	50.73333333	1
84	0	39.93333333	1
85	0	160.9666667	0
86	0	34.03333333	1
87	0	54.46666667	1
88	0	46.2	1
89	0	137.6	0
90	1	25.93333333	1
91	1	130.1	0
92	1	41.23333333	1
93	0	33.1	1
94	0	108.8	0
95	1	57	1
96	1	73.3	1
97	0	171.2	0
98	1	165.6333333	0
99	0	48.9	1
100	0	68.66666667	1
101	0	50.33333333	1
102	1	176.1	0
103	0	95.06666667	1
104	1	37.6	1
105	0	85.06666667	0
106	0	34.33333333	1
107	0	221	0
108	1	90	0
109	1	88	0
110	1	82	0
111	1	23	1
112	0	52	1
113	0	75	0
114	1	49	1
115	1	76	0
116	0	72	0

117	1	64	0
118	1	67	0
119	1	60	0
120	1	81	0
121	0	34	1
122	0	78	0
123	1	69	0
124	1	59	0
125	1	61	0
126	1	77	0
127	1	69	1
128	0	13	1
129	1	80	0
130	0	70	0
131	1	62	0
132	1	76	0
133	0	68	1
134	1	23	1
135	1	48	1
136	0	35	1
137	1	72	1
138	1	66	0
139	1	65	0
140	0	4	0
141	1	88	0
142	0	52	1
143	1	59	1
144	1	76	0
145	1	15	1
146	1	31	1
147	1	75	0
148	1	79	0
149	1	69	1
150	1	65	0
151	1	39	1
152	0	42	1
153	1	62	0
154	1	73	0
155	1	11	1

156	1	35	0
157	1	46	1
158	1	1	0
159	1	70	0
160	1	76	1
161	1	63	1
162	1	60	0
163	1	62	0
164	1	50	0
165	1	38	1
166	1	12	1
167	1	59	0
168	1	12	1
169	0	37	1
170	1	24	1
171	1	63	0
172	1	69	0
173	1	66	0
174	0	66	0
175	1	55	0
176	1	59	0
177	1	10	1
178	0	23	1
179	1	93	0
180	1	52	0
181	1	63	0
182	1	25	1
183	1	57	1
184	1	36	1
185	0	38	1
186	0	65	0
187	1	37	0
188	1	8	1
189	1	22	1
190	1	39	0
191	1	7	1
192	1	62	0
193	1	47	1
194	1	41	0

195	1	18	1
196	1	35	1
197	0	32	0
198	1	60	0
199	1	80	0
200	1	70	0
201	0	131	0
202	1	6	0
203	1	48	1
204	1	18	0
205	1	94	0
206	1	72	0
207	1	21	0
208	1	9	0
209	1	60	0
210	1	15	0
211	0	2	0
212	1	16	0
213	1	10	1
214	1	61	0
215	0	1	0
216	1	41	0
217	1	18	1
218	1	23	1
219	0	19	1
220	1	89	0
221	0	7	1
222	1	50	0
223	1	62	0
224	1	7	1
225	1	33	0
226	0	15	0
227	1	91	1
228	0	15	1
229	1	54	0
230	0	14	1
231	1	4	1
232	1	99	0
233	1	1	1

234	1	50	0
235	0	8.52	0
236	0	8.23	0
237	0	0.03	1
238	0	8.6	0
239	1	1.59	1
240	1	6.09	1
241	0	0.19	1
242	0	6.93	0
243	0	7.71	0
244	1	0.9	1
245	1	3.74	1
246	0	7.2	0
247	0	5.87	0
248	1	5.42	0
249	0	5.03	0
250	1	2.28	1
251	0	1.86	1
252	0	2.82	1
253	0	4.46	0
254	1	3.95	0
255	0	3.51	0
256	0	4.89	0
257	0	4.76	1
258	0	6.74	0
259	1	5.38	0
260	1	2.04	1
261	1	6.03	1
262	1	127	0
263	0	12.57	1
264	1	0.67	1
265	1	98.93	0
266	0	87.7	1
267	0	10.33	1
268	1	4.87	1
269	0	20.37	1
270	0	66.47	1
271	1	110.8	0
272	0	22.57	1

273	0	103.7	0
274	0	124.83	0
275	1	21.3	1
276	0	0.87	1
277	0	8.27	1
278	1	116.83	0
279	0	17.3	1
280	0	79.87	1
281	1	16.7	1
282	0	24.17	1
283	0	46.7	1
284	1	2.53	1
285	0	63.87	0
286	0	27.97	0
287	0	89.73	0
288	0	110.8	0
289	1	20.53	1
290	0	67.87	0
291	0	20.93	0
292	0	23.13	0
293	1	20.37	1
294	0	5.53	1
295	0	13.73	1
296	1	124.3	0
297	1	89.03	0
298	1	89.03	0
299	0	78.63	0
300	0	60.73	1
301	0	2.5	1
302	0	7.13	1
303	0	59.83333333	0
304	0	11.6	1
305	0	34.8	0
306	0	35.66666667	0
307	0	43.36666667	0
308	1	60.76666667	0
309	1	33.8	1
310	0	58.53333333	0
311	1	33.56666667	1

312	0	24.1	0
313	0	39.6	1
314	0	35.2	0
315	0	42.8	1
316	1	68.33333333	0
317	0	16.93333333	1
318	1	20.3	1
319	0	73.33333333	0
320	0	63.53333333	0
321	1	21.33333333	1
322	0	54.3	0
323	0	22.56666667	1
324	0	19.16666667	1
325	0	49.5	0
326	1	49.43333333	1
327	0	4.9	1
328	0	12.46666667	1
329	0	70.86666667	0
330	0	1.066666667	1
331	0	55.36666667	1
332	1	42.63333333	0
333	0	59.46666667	0
334	1	11.16666667	1
335	0	27.2	1
336	0	28.36666667	1
337	0	51.23333333	0
338	0	12.33333333	0
339	0	37.5	0
340	0	29.13333333	1
341	0	1.9	1
342	0	24.33333333	1
343	1	41.66666667	0
344	1	41.63333333	0
345	0	24.13333333	1
346	1	19.83333333	1
347	1	39.83333333	0
348	1	6.5	1
349	0	39	1
350	1	7	1

351	1	48	0
352	0	31	0
353	1	6	0
354	0	3	1
355	1	2	1
356	0	24.5	1
357	0	11	1
358	0	9	1
359	0	27	0
360	1	10.5	1
361	0	27.17043121	0
362	0	33.83983573	0
363	0	34.7597536	0
364	0	25.56057494	0
365	0	32.65708418	0
366	0	12.12320328	1
367	0	31.08008214	0
368	0	42.38193018	1
369	0	39.09650924	0
370	0	35.38398358	0
371	0	40.21355236	1
372	0	26.34907597	0
373	0	49.7412731	0
374	0	40.7063655	0
375	0	40.60780288	0
376	0	32.95277207	1
377	0	50.1026694	0
378	0	24.24640657	0
379	0	32.42710472	0
380	0	5.585215608	0
381	0	7.786447644	0
382	0	11.36755646	0
383	0	35.67967146	0
384	0	46.42299794	0
385	0	26.0862423	1
386	0	25.95482546	1
387	0	58.02053388	0
388	0	28.8788501	1
389	0	43.07186858	1

390	0	69.71663244	0
391	0	121	0
392	0	21	1
393	0	75	0
394	0	165	0
395	1	68	0
396	0	85	0
397	0	87	0
398	1	51	0
399	1	34	1
400	1	20	1
401	0	43	0
402	1	29	1
403	1	58	0
404	0	102	0
405	0	8	1
406	0	89	1
407	1	131	0
408	0	101	0
409	0	25	1
410	0	67	0
411	0	83	0
412	0	96	0
413	1	1	0
414	0	36	0
415	0	175	1
416	0	182	0
417	1	32	0
418	0	74	1
419	1	62	0
420	1	25	1
421	0	90	1
422	1	40	1
423	0	85	0
424	0	113	0
425	0	12	1
426	0	72	0
427	1	42	0
428	0	60	0

429	1	52	1
430	0	72	0
431	0	14	1
432	0	13	1
433	0	0	1
434	0	153	0
435	0	18	0
436	0	38	1
437	1	150	1
438	0	28	1
439	1	76	0
440	1	127	1
441	1	18	1
442	0	108	0
443	0	43	0
444	0	142	0
445	0	127	0
446	0	175	1
447	0	57	0
448	0	61	0
449	1	65	0
450	0	104	0
451	0	115	0
452	1	2	1
453	1	13	1
454	1	14	1
455	1	134	0
456	0	0	1
457	0	11	1
458	0	74	0
459	1	29	0
460	0	163	0
461	1	107	1
462	0	99	1
463	0	21	1
464	0	4	1
465	0	23	1
466	0	17	1
467	0	103	1

468	0	42	1
469	0	55	1
470	0	179	1
471	0	23	1
472	0	174	0
473	0	26	1
474	0	3	0
475	0	14.56666667	1
476	1	24.76666667	0
477	0	86.7	0
478	0	39.1	0
479	0	78.96666667	0
480	1	62.63333333	0
481	1	20.2	0
482	1	10	1
483	0	63.96666667	0
484	1	61.16666667	0
485	0	60.93333333	0
486	0	61.36666667	1
487	1	21.3	1
488	0	43.7	1
489	0	36.6	0
490	0	39.63333333	0
491	0	128.7666667	0
492	0	49.16666667	0
493	0	61.86666667	0
494	1	102.2333333	0
495	1	38.06666667	0
496	1	60.83333333	0
497	0	53.66666667	0
498	1	67.13333333	0
499	0	60.86666667	0
500	1	73.03333333	0
501	1	111.4	0
502	0	44.26666667	0
503	1	97.5	0
504	0	96	0
505	0	57.46666667	0
506	1	92.16666667	0

507	0	50.96666667	0
508	1	7.366666667	1
509	1	18.2	1
510	0	11.53333333	1
511	1	28.43333333	1
512	1	88.7	1
513	0	27.23333333	0
514	0	14.16666667	1
515	1	42.7	0
516	1	34.8	0
517	0	18.83333333	0
518	0	35.3	1
519	1	54.16666667	1
520	1	56.5	1
521	1	99.2	0
522	0	38.1	1
523	1	56.06666667	0
524	0	41.4	1
525	1	75.06666667	0
526	1	31.43333333	1
527	1	44.86666667	1
528	0	49.4	1
529	1	79.53333333	0
530	1	52	0
531	1	33.36666667	1
532	0	8.633333333	1
533	0	76.3	0
534	0	63.86666667	0
535	1	68.26666667	0
536	0	49.16666667	0
537	1	51.3	0
538	1	21.2	0
539	0	42.5	0
540	1	66.83333333	0
541	1	65.5	0
542	1	80.83333333	0
543	1	52.2	0
544	1	33	0
545	1	24.3	0

546	1	78.46666667	0
547	1	27.26666667	0
548	0	66.96666667	0
549	1	84.96666667	0
550	0	113.7	0
551	0	60.2	0
552	0	64.56666667	0
553	1	50.66666667	0
554	1	45.83333333	0
555	1	58.53333333	0
556	1	53.23333333	0
557	1	57.13333333	0
558	1	54.56666667	0
559	1	55.1	0
560	1	39.86666667	0
561	1	56.73333333	0
562	0	68.33333333	0
563	1	41.86666667	0
564	1	27.26666667	0
565	0	38.46666667	0
566	1	101.9333333	0
567	0	64.7	0
568	0	53.2	0
569	1	81.66666667	0
570	1	62.7	0
571	0	54.56666667	0
572	1	67.8	0
573	1	71.13333333	0
574	1	25.96666667	1
575	1	18	1
576	1	43.9	0
577	1	66	0
578	0	23.4	1
579	0	18.8	1
580	1	26.26666667	1
581	1	48.76666667	0
582	0	44.83333333	1
583	1	27.73333333	0
584	1	89.56666667	0

585	0	37.4	1
586	1	88.93333333	0
587	1	64.53333333	0
588	1	29.03333333	0
589	1	89.03333333	0
590	0	49.4	0
591	1	37.6	0
592	1	34.73333333	1
593	0	40.96666667	1
594	1	75.43333333	1
595	1	59	0
596	1	47.76666667	1
597	1	60.76666667	0
598	1	66.26666667	0
599	0	47.8	0
600	1	21	1
601	1	51.23333333	0
602	0	47.63333333	1
603	0	34.6	1
604	1	82.86666667	0
605	1	24.66666667	0
606	1	34.73333333	0
607	0	42.23333333	0
608	0	33.9	0
609	0	34.7	0
610	1	61.96666667	0
611	1	57.23333333	0
612	1	49.8	0
613	1	25.13333333	0
614	1	78.8	0
615	0	57.76666667	0
616	1	64.7	0
617	1	25.8	0
618	1	97.1	0
619	1	60.1	0
620	1	63.73333333	0
621	1	49.36666667	0
622	1	41.03333333	0
623	1	65.46666667	0

624	1	111.7333333	0
625	1	71.93333333	0
626	1	115.5333333	0
627	1	108.7666667	0
628	1	60.76666667	0
629	0	61.86666667	0
630	1	81.63333333	0
631	1	61.96666667	0
632	1	66.6	0
633	0	61.73333333	0
634	1	63.13333333	0
635	1	62.03333333	0
636	1	55.63333333	0
637	0	106.3	0
638	0	60.73333333	0
639	1	60.3	0
640	1	85.06666667	0
641	1	98.93333333	0
642	1	102.2	0
643	0	47.06666667	0
644	1	43.9	0
645	0	39.33333333	0
646	1	56.26666667	0
647	1	47.23333333	0
648	0	47.53333333	0
649	0	55.8	0
650	0	61.6	0
651	1	61.86666667	0
652	0	59.33333333	0
653	1	69.46666667	0
654	1	62.63333333	0
655	0	37.13333333	0
656	1	93.53333333	0
657	1	55.43333333	0
658	1	57.53333333	0
659	0	54.3	0
660	1	72.63333333	0
661	1	63.9	0
662	0	48.2	0

663	1	38.73333333	0
664	0	41.03333333	0
665	1	31.36666667	0
666	1	54.1	0
667	1	61.83333333	0
668	1	46.8	0
669	1	74.03333333	0
670	1	28.3	0
671	1	27.7	0
672	1	107.3	0
673	0	86.16666667	0
674	1	81.4	0
675	1	63.66666667	0
676	1	61.13333333	0
677	0	97.26666667	0
678	1	76.16666667	0
679	0	71.63333333	0
680	1	85.36666667	0
681	1	64.9	0
682	1	61.36666667	0
683	1	65.7	0
684	1	80.3	0
685	1	54.13333333	0
686	0	81	0
687	1	61.86666667	0
688	1	50.53333333	0
689	1	60.63333333	0
690	0	80.43333333	0
691	0	61.63333333	0
692	0	72.7	0
693	1	72.26666667	0
694	1	71.8	0
695	1	69.1	0
696	1	66.46666667	0
697	1	71.23333333	0
698	1	66.6	0
699	0	63.6	0
700	1	55.33333333	0
701	0	48	0

702	1	39	0
703	0	21	1
704	1	15	1
705	0	33	0
706	0	44	0
707	0	20	1
708	0	47	0
709	0	39	0
710	0	61	0
711	0	20	1
712	0	42	0
713	0	43	0
714	0	52	1
715	0	45	0
716	1	34	1
717	0	3	1
718	0	44	0
719	0	50	0
720	0	10	1

C: Pair-wise comparison

Group		High KDM1A + Low TIMP3	High KDM1A + High TIMP3	Low KDM1A + Low TIMP3	Low KDM1A + High TIMP3
		Significance	Significance	Significance	Significance
**Log Rank (Mantel-Cox)**	High KDM1A + Low TIMP3		.063288	.531955	.000021
	High KDM1A + High TIMP3	.063288		.340296	.020578
	Low KDM1A + Low TIMP3	.531955	.340296		.000403
	Low KDM1A + High TIMP3	.000021	.020578	.000403	

(A, B) Patient survival data based on KDM1A (**A**) or TIMP3 (**B**) expression. (**C**) Logrank test for the pair-wise comparison among four groups in patient survival analysis. Four groups are: NSCLC patients with high KDM1A and low TIMP3 expressions, high KDM1A and high TIMP3 expressions, low KDM1A and low TIMP3 expressions, and low KDM1A and high TIMP3 expressions. The data were downloaded from http://kmplot.com/ [1].
